# Comparative Effectiveness of Pharmacological and Non-Pharmacological Interventions for Nausea and Vomiting in Pregnancy: A Systematic Review and Network Meta-Analysis

**DOI:** 10.3390/nu18081293

**Published:** 2026-04-20

**Authors:** Lőrinc Frivaldszky, Mahmoud Obeidat, Péter Hegyi, Csongor Kárpáti, Zoltán Kobza, Nándor Ács, Ferenc Bánhidy, Gergely Agócs, Boglárka Lilla Szentes, Márton Keszthelyi

**Affiliations:** 1 Centre for Translational Medicine, Semmelweis University, Baross utca 22, H-1085 Budapest, Hungary; frivaldszky.lorinc@stud.semmelweis.hu (L.F.); dr.mahmoud.obeidat@gmail.com (M.O.); hegyi.peter@semmelweis.hu (P.H.); karpati.csongor@stud.semmelweis.hu (C.K.); kobza.zoltan@stud.semmelweis.hu (Z.K.); acs.nandor@semmelweis.hu (N.Á.); banhidy.ferenc@semmelweis.hu (F.B.); agocs.gergely@semmelweis.hu (G.A.);; 2 MRE Bethesda Children’s Hospital, Bethesda utca 3, H-1146 Budapest, Hungary; 3 Department of Obstetrics and Gynecology, Semmelweis University, Üllői út 78/A, H-1082 Budapest, Hungary; 4 Institute for Translational Medicine, Medical School, University of Pécs, Szigeti út 12, H-7624 Pécs, Hungary; 5 Institute of Pancreatic Diseases, Semmelweis University, Tömő utca 25-29, H-1083 Budapest, Hungary

**Keywords:** nausea and vomiting in pregnancy, vitamin B6, doxylamine, ginger, acupuncture, acupressure, network meta-analysis

## Abstract

**Background:** Nausea and vomiting in pregnancy affects up to 80% of pregnant women and may progress to hyperemesis gravidarum, leading to maternal morbidity and adverse pregnancy outcomes. Despite numerous pharmacological and non-pharmacological options, the comparative efficacy and safety of these interventions remain unclear. **Methods:** We conducted a systematic review and network meta-analysis of randomized controlled trials assessing pharmacological and non-pharmacological interventions for nausea and vomiting in pregnancy. The databases searched included CENTRAL, PubMed, and EMBASE (up to 28 May 2024). Eligible trials compared interventions with a placebo in pregnant women with nausea and vomiting in pregnancy. The primary outcomes were symptom severity, assessed using validated tools. Safety outcomes included adverse effects. Data were pooled using frequentist pairwise and network meta-analyses. The risk of bias was assessed using the RoB2 tool, and the certainty of evidence was evaluated using the CINeMA framework. **Results:** Of 9844 records screened, 24 randomized controlled trials (3017 participants) met the inclusion criteria, encompassing 16 intervention categories. Network analysis ranked quince, vitamin B6 with pomegranate and mint, acupressure P6, dimenhydrinate, and acupuncture combined with doxylamine–pyridoxine as the most effective interventions for reducing symptoms of nausea and vomiting in pregnancy, with considerable uncertainty and low-to-moderate quality of evidence. Reporting of adverse events was limited. Risk of bias was low to moderate. **Discussion:** Most interventions demonstrated significant benefit over a placebo. However, high heterogeneity and sparse reporting of adverse effects warrant caution when translating these results into clinical practice. **Conclusions:** This study indicates that both pharmacological (vitamin B6, metoclopramide, dimenhydrinate) and non-pharmacological (ginger, quince, acupressure, acupuncture) interventions might be effective in reducing symptoms of nausea and vomiting in pregnancy.

## 1. Introduction

Nausea and vomiting in pregnancy (NVP) is a common condition affecting up to 80% of pregnant women, with symptoms typically emerging between the 4th and 9th weeks of gestation and often resolving by the end of the first trimester [1]. While NVP is generally self-limiting, it can range from mild nausea to severe, persistent vomiting known as hyperemesis gravidarum (HG), which may lead to dehydration, electrolyte imbalances, and weight loss, potentially requiring hospitalization [2]. The condition not only affects maternal health but also has significant psychosocial and economic consequences, including reduced quality of life, increased healthcare utilization, and loss of productivity [3]. In severe cases, NVP has been associated with adverse pregnancy outcomes such as low birth weight and preterm birth, emphasizing the need for effective management strategies [2].

Current treatment options for NVP include dietary and lifestyle modifications, pharmacologic therapies, and complementary approaches such as acupuncture and ginger supplementation [4]. First-line pharmacologic treatments often include pyridoxine (vitamin B6) and doxylamine, which are effective and safe for managing mild to moderate symptoms [5,6]. For more severe cases, antiemetics such as ondansetron, metoclopramide, and promethazine may be prescribed, although concerns regarding their safety profiles persist [7]. Non-pharmacologic interventions, including acupuncture, hypnosis, and cognitive behavioral therapy, have been explored with varying degrees of success [4]. Despite the variety of available interventions, there remains no universally accepted treatment protocol, and many pregnant women continue to suffer from inadequately managed symptoms.

A significant gap exists in our understanding of the safety and efficacy of pharmacologic and non-pharmacologic interventions for NVP, particularly regarding fetal outcomes and long-term maternal health. Concerns about teratogenicity and adverse pregnancy outcomes have led to inconsistent prescribing practices and reluctance among healthcare providers to use certain medications [7]. Additionally, research on the comparative effectiveness of different treatments remains limited, with few high-quality randomized controlled trials (RCTs) assessing the optimal management strategies for NVP across diverse populations. More robust evidence is needed to establish clear guidelines and improve patient-centered care.

Previous systematic reviews and meta-analyses have synthesized available data on the effectiveness and safety of NVP treatments. Yet, they often report heterogeneous findings due to differences in study design, sample size, and outcome measures [4]. While some reviews suggest a benefit of ginger and acupressure in mild cases, others highlight insufficient data to draw definitive conclusions on the long-term safety of pharmacological treatments [8]. Additionally, many systematic reviews focus on a limited subset of interventions, leaving gaps in comparative effectiveness research. A comprehensive evaluation of existing evidence is necessary to guide clinical practice and inform future research priorities in NVP management.

## 2. Materials and Methods

This work was carried out as part of the Systems Education Program at Semmelweis University [9] and conducted within the Translational Medicine (TM) Cycle Framework by the Academia Europaea [10].

We report our systematic review and meta-analysis based on the recommendations of the Preferred Reporting Items for Systematic reviews and Meta-Analyses (PRISMA) 2020 guidelines [11] (Supplementary Table S1), while following the Cochrane Handbook [12]. The study protocol was registered on PROSPERO (CRD42024548049), and we fully adhered to its guidelines [13,14].

### 2.1. Eligibility Criteria, Information Sources and Search Strategy

The study followed a population–intervention–control–outcome (PICO) [15] framework—population: pregnant women with NVP; intervention: different pharmacological and non-pharmacological interventions against NVP; control: placebo; outcome: efficacy measures [Pregnancy-Unique Quantification of Emesis (PUQE) scores—assessing the severity of nausea and vomiting symptoms; Visual-Analog Scale (VAS) scores—assessing subjective symptom severity; Nausea and Vomiting in Pregnancy Quality Of Life (NVPQOL) scores—assessing the impact of symptoms on quality of life; Rhodes-index scores, number of vomiting episodes] and adverse effects. Studies reported different pharmacological and non-pharmacological interventions for NVP, comprising RCTs. Excluded were cohort studies, cross-sectional studies, non-randomized controlled trials, case series, case reports, animal studies, conference abstracts, and reviews.

Our systematic search was conducted in Cochrane Central Register of Controlled Trials (CENTRAL), Medline (via PubMed), and Embase on the 28 May 2024. No filters or other restrictions were applied. For detailed search terms (see Supplementary S1). Our search key consisted of three domains regarding clinical condition, population, and study type.

### 2.2. Selection Process

Two independent reviewers (ZK and CK) screened titles and abstracts for eligibility using EndNote (EndNote 20, 2013, Clarivate, Philadelphia, PA, USA) and Rayyan software [16]. Discrepancies were resolved by a third reviewer (LF), and Cohen’s kappa coefficient was reported to assess inter-rater agreement.

### 2.3. Data Collection Process and Data Items

Data selection and extraction were performed by two independent reviewers (ZK and CK) using Excel (Office 365, Microsoft, Redmond, WA, USA), with a third reviewer (FL) resolving disagreements. Extracted data included: (1) study characteristics: author, year, country of origin, population details (number, age, BMI, parity, gestational week); (2) intervention details (intervention type, dosage, time) and other drugs; (3) primary outcomes: PUQE, VAS, NVPQOL, Rhodes-index scores, number of vomiting episodes; and (4) safety outcomes: adverse effects. Data from visual sources were estimated using WebPlotDigitizer software [17]. All scales used in our final analysis (PUQE, VAS, Rhodes-index) were subjective assessment questionnaires measuring the intensity of NVP symptoms, with lower scores indicating less severe symptoms and higher scores indicating more severe symptoms. Minimum and maximum of scores were: PUQE (3–15), VAS (0–10), Rhodes-index (0–32) [18,19,20,21].

### 2.4. Study Risk-of-Bias Assessment

The risk of bias was assessed using the RoB2 tool and visualized with the Risk-of-bias VISualization (ROBVIS) tool [12,22,23]. Two reviewers (LF and CK) conducted assessments, with a third reviewer (MO) resolving disagreements. The CINEMA (Confidence in Network Meta-Analysis) assessment was used to evaluate the certainty of evidence in network meta-analyses [24,25].

### 2.5. Synthesis Methods

The different scales used to report nausea and vomiting scores shared similar definitions of minimum and maximum scores, which made it possible to harmonize all scores onto a 0–10 scale, where 0 indicated no discomfort and 10 the worst nausea imaginable.

This enabled the use of mean differences (MDs) as the effect size for antiemetic treatment efficiency. Also, we grouped the reduction in nausea scores by follow-up time into “one-week follow-up” and “one-month follow-up”.

Control conditions were categorized as placebo, sham intervention, or no intervention and modeled as separate nodes, as these comparators were considered to differ in both contextual (expectation-related) and potential physiological effects.

The inclusion of only RCTs allowed us to perform a network meta-analysis using a frequentist approach. Network meta-analysis enables the comparison of multiple interventions by integrating direct and indirect evidence across a connected network of interventions. For each pairwise comparison, we calculated MDs and their 95% confidence intervals (CIs). We used random-effects modeling since we expected considerable between-study heterogeneity. The variance component was estimated using restricted maximum likelihood (REML).

To evaluate the transitivity assumption, we systematically compared the distribution of key clinical and methodological effect modifiers—including symptom severity, gestational age, and intervention protocols—across all included studies. This qualitative assessment ensured that the studies were sufficiently comparable in terms of population characteristics, interventions, and outcomes, thereby supporting the validity of indirect comparisons in our network meta-analysis.

Multi-arm studies were included using multi-arm correction methods to ensure accurate variance estimates. Treatments were ranked using the P-score metric, which quantifies the certainty that an intervention is better than another based on point estimates and standard errors [26].

Diagnostics for consistency and reliability included netsplit analysis, direct evidence proportion plots, minimal parallelism plots, and mean path length plots. These methods evaluated the robustness of the effect size estimates by distinguishing between direct and indirect evidence. Small study publication bias was assessed using funnel plots and a modified Egger’s test.

The results were visualized using forest plots (where interventions are compared to a reference, in our case, “placebo”), league tables (where the upper triangle shows direct pairwise comparisons, while the lower triangle shows the results of network meta-analysis), network graphs (where circles at the vertices represent intervention arms, and edges represent direct comparisons), and rank plots (where treatments are ranked based on the aforementioned P-score metric), providing a comprehensive summary of the intervention comparisons. Netpairwise plots were used to visualize all direct comparisons, with pooled estimates for comparisons involving at least three studies. All analyses were performed in R using the meta [27], netmeta [28], and dmetar [29] packages.

## 3. Results

### 3.1. Study Selection

**Figure 1:**
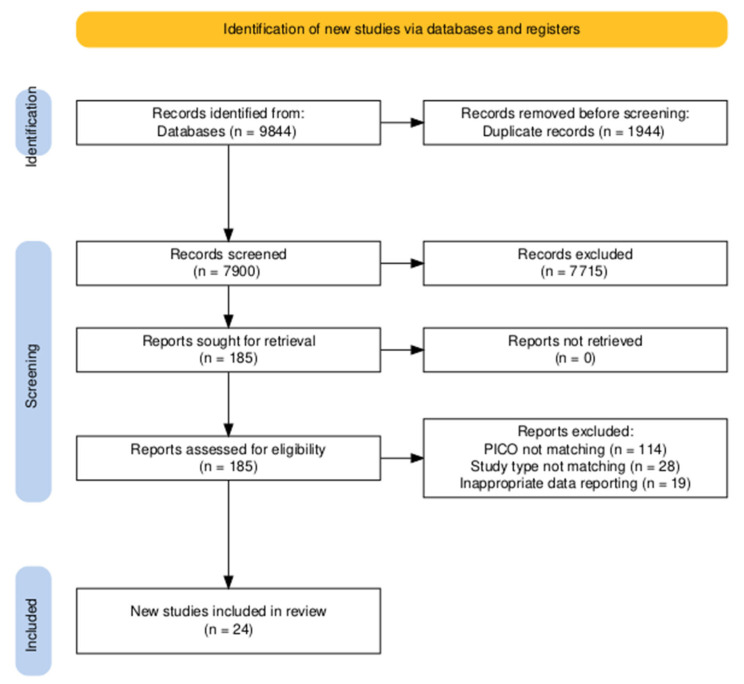
PRISMA flowchart of the selection process.

A total of 9844 studies were identified in our systematic search. After duplicate removal, 7900 records were screened based on the title and abstract. We excluded 7715 records and retrieved 185 reports to be assessed based on their full texts. Out of 185 reports, we found 24 eligible articles; 161 were excluded, mainly due to not matching PICO, ineligible study type, or inappropriate data reporting. A summary of the selection process is shown in Figure 1. Cohen’s kappa values were 0.91 and 0.99.

### 3.2. Study Characteristics

Our review encompassed 24 studies involving a total of 3017 patients. Study arms were grouped by their intervention types into the following 16 intervention categories: B6 [30,31,32,33,34,35,36], B6 and pomegranate and mint [30], acupressure KID21 [37], acupressure P6 [35,37,38,39,40,41], acupuncture and doxylamine–pyridoxine [42], acupuncture [42,43,44,45], dimenhydrinate [31], doxylamine–pyridoxine [42,46,47], essential oil [48,49,50,51], ginger [32,34,36,40,46,52,53], metoclopramide [53], quince [33], no intervention [45], placebo [34,36,38,40,41,43,44,47,48,49,50,51,52,53], sham acupressure [39], and sham acupuncture [42,45]. Included studies originated from Iran (14), Australia (1), India (1), Malaysia (1), Brazil (1), Turkey (2), Thailand (2), Canada (1), and China (1). All studies included patients in the first and the early second trimester, as NVP symptoms are most prevalent in this timeframe. The duration of most treatments ranged from 3 to 7 days; in exceptional cases, interventions lasted up to 2 to 3 weeks. Further study characteristics, including countries of origin, assessment scale preferences, number of included patients, treatment durations, intervention details, drug dosages, BMI values, parity, and gestational age of included patients, are detailed in Supplementary Table S2.

### 3.3. Risk-of-Bias Assessment

Most of the outcomes of the studies included in the meta-analysis were rated as having a low or moderate risk of bias. Most concerns emerged in the domains related to outcome measurement and reporting, mainly due to the subjective matter of the measurement scales used. The risk-of-bias assessment for our primary outcome is shown in Figure S1.

### 3.4. Efficacy Assessment

**Figure 2:**
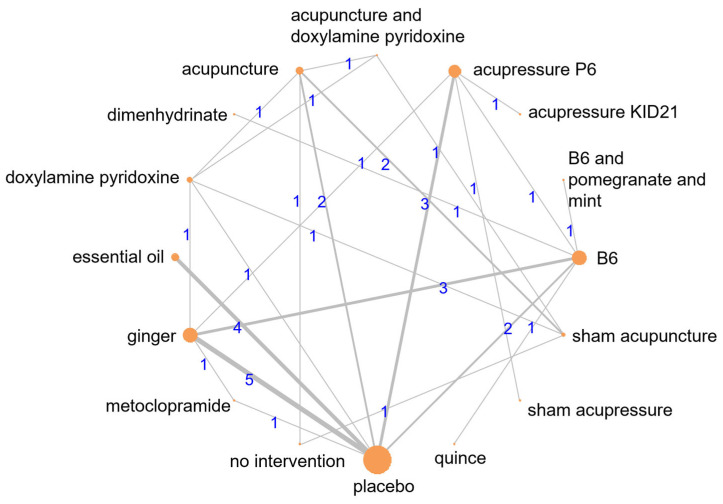
Network plot of efficacy analysis. The numbers above the connecting lines indicate the count of studies comparing the two linked interventions, while the size of each node (dot) reflects the total number of studies associated with that specific intervention.

**Figure 3:**
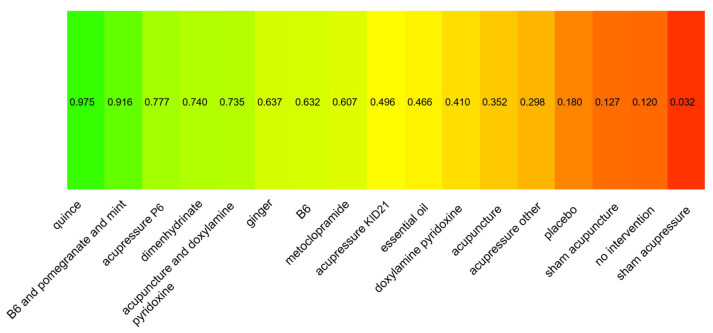
P-scores of interventions—efficacy assessment.

**Figure 4:**
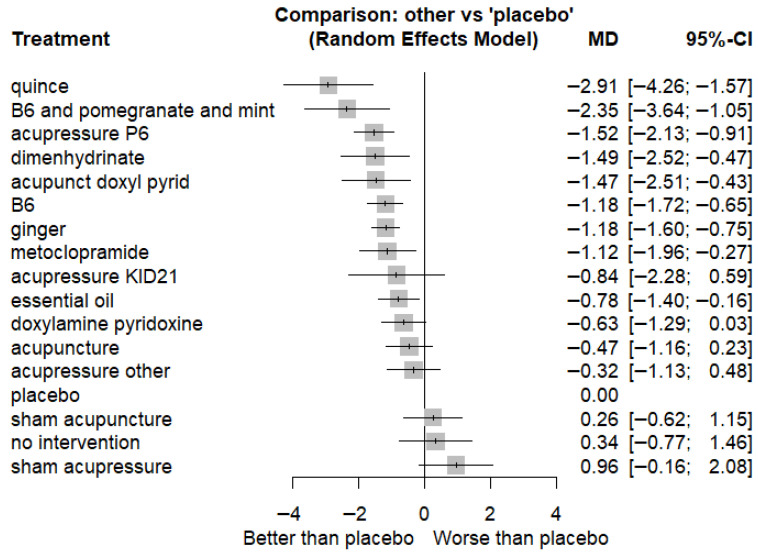
Forest plot with placebo reference—mean differences in NVP symptom score reductions. MD: mean differences; CI: confidence interval.

Our primary outcome analysis results include a network of 24 studies, including 18 two-arm studies and six multi-arm studies (see Figure 2). Outcome data were reported at 1-week follow-up and standardized to a scale of 0–10. The total number of patients examined in the network was 3017. P-scores indicated that quince administration is likely to result in the most considerable reduction in NVP scores, followed by B6 and pomegranate and mint, acupressure P6, dimenhydrinate, and acupuncture and doxylamine–pyridoxine (see Figure 3). The league table for the primary outcome (Table 1) shows the pairwise comparisons of different preparation techniques; most comparisons were results of indirect estimates, meaning that the ranking of different interventions should be interpreted with caution. Compared to the placebo, most included interventions showed a statistically significant reduction in NVP scores (see Figure 4). The consistency analysis showed that the comparisons in the network were consistent (Figure S2). Further sensitivity analyses were conducted excluding single-study nodes and digitized outcomes or based on SMD calculations to ensure robustness (see Supplementary Figures S4–S8). Regarding 1-month follow-up data, no network meta-analysis could be conducted because the data was particularly sparse, even compared with 1-week follow-up outcomes.

### 3.5. Safety Assessment

The reporting of safety outcomes was sparse in the included studies. Thus, no meta-analysis could be performed for this outcome. Overall, studies have found low occurrence of adverse effects, especially in the case of non-pharmacological interventions (see Supplementary Table S3). Patients taking ginger supplements reported loose stools (2.94%) [46], heartburn (12.1%), sedation (11.1%), and arrhythmia (1.6%) [32], as well as dizziness (3.12%) and heartburn (3.2%) [52]. Regarding the administration of doxylamine–pyridoxine, hyperacidity (6.89%) was reported [46]. Certain patients receiving B6 supplements experienced heartburn (3.2%), sedation (17.5%), headache (3.1%) [32], or i drowsiness (4.46%) [31]. One study reported that 52.94% of patients taking dimenhydrinate experienced drowsiness [31]. Acupressure was associated with irritation in one case (3.03%) [35]. Patients inhaling peppermint oil reported headache (3.57%), dizziness (1.79%), and shortness of breath (1.79%) in one study [48].

### 3.6. Publication Bias and Heterogeneity

Publication bias was assessed using comparison-adjusted funnel plots, which are recommended in the context of network meta-analysis to account for multiple comparisons and differing reference groups. However, several important limitations should be considered when interpreting these plots. First, visual assessment of funnel plot asymmetry is inherently subjective and may be particularly challenging in complex networks. Second, comparison-adjusted funnel plots may be difficult to interpret when the number of studies per comparison is small, as is the case in parts of our network, limiting their ability to reliably detect small-study effects. Third, observed asymmetry may arise not only from publication bias but also from clinical or methodological heterogeneity, differences in study precision, or chance. However, the funnel plot showed no asymmetry. A potential source of further publication bias may stem from the limited coverage of regional databases, especially for Chinese and Arabic literature. Heterogeneity was relatively high for all outcomes due to differences in baseline characteristics of study participants (tau-squared: 0.1774, network-level I-squared: 68.6% (95% CI: 47.3%; 81.3%), differences in interventions, or differences in definitions and measurements of outcomes.

### 3.7. Certainty of Evidence

Using the CINeMA tool, the overall quality of evidence was rated as low to moderate. The major data quality concerns for all outcomes were due to high imprecision, incoherence, and heterogeneity. Imprecision was reflected in the wide range of CIs and the limited number of patients in certain studies. Additionally, incoherence arose from significant heterogeneity in certain methodological and clinical domains across the reported studies. The result of the certainty-of-evidence analysis is detailed in Supplementary Table S4.

## 4. Discussion

### 4.1. Principal Findings

This systematic review and network meta-analysis evaluated the efficacy of both pharmacological and non-pharmacological interventions for NVP. The analysis synthesized data from 24 RCTs, encompassing 3017 participants. The findings highlighted the effectiveness of specific dietary supplements, such as vitamin B6, quince, ginger, mint, and pomegranate, as well as acupressure and acupuncture in alleviating NVP symptoms. Among pharmacological treatments, dimenhydrinate, doxylamine–pyridoxine, and metoclopramide, often combined with non-pharmacological approaches, also demonstrated clinically meaningful benefits. However, ranking these interventions remains challenging due to high heterogeneity, limited study and participant numbers, and insufficient safety data, which collectively hinder definitive comparative conclusions. Further uncertainty arises from differences in baseline symptom severity, age, BMI, gestational age, intervention duration, and treatment dosage regimens, as these factors may affect treatment outcomes, particularly when ranking interventions. Certain interventions might have different effects depending on baseline symptom levels, treatment durations, and dosages, while age and BMI might influence the pharmacodynamics and pharmacokinetics of drugs and dietary supplements, and even modify how non-pharmacological interventions affect NVP symptoms.

### 4.2. Comparison with the Existing Literature

The American College of Obstetricians and Gynecologists (ACOG) practice bulletin recommends vitamin B6 alone or combined with doxylamine as a safe and effective first-line pharmacotherapy for NVP [6]. Our findings align with these guidelines, demonstrating clinically relevant improvements in NVP symptoms following the administration of combined doxylamine–pyridoxine. Additionally, the existing literature consistently supports the safety profile of doxylamine–pyridoxine and doxylamine alone, indicating no increased risk of adverse pregnancy outcomes [54,55,56]. Some studies further suggest that specific delayed-release formulations of this combination may offer the best efficacy in managing NVP [54]. Multiple systematic reviews and meta-analyses also recognize vitamin B6 administration as a safe and effective treatment for NVP [57,58]. Metoclopramide is effective in reducing symptoms of NVP; however, it is associated with a high rate of adverse effects—31% of pregnant women reported discontinuing its use due to these side effects [59]. Dimenhydrinate was also associated with improved NVP symptoms, even when compared to vitamin B6 or ginger [31,60].

Several reviews suggest that ginger may help reduce nausea symptoms during pregnancy, though the evidence is limited by the small number of studies, inconsistent outcome reporting, and overall low quality [58,61,62,63]. Our findings also support the beneficial effects of ginger. However, the variability in administration methods and dosages of ginger supplements poses challenges to standardizing treatment regimens. Importantly, according to the available literature, ginger has not been associated with side effects or adverse events in pregnancy [61]. Based on the evidence from these systematic reviews, ginger could be considered a safe and potentially effective alternative for women experiencing NVP, even compared to other interventions such as vitamin B6 administration [58,63,64]. Essential oils and other dietary supplements, such as quince, pomegranate, or mint products, have also been evaluated as safe and effective interventions for managing NVP [65,66,67]. However, regarding inhalative agents, different products and delivery methods can result in different outcomes; thus, further research is needed in this area to inform clinical choices. Lastly, it is important to note that the availability of certain dietary supplements or products is highly influenced by geographical or cultural factors; thus, practitioners must take these factors into account when interpreting study results for clinical practice.

A substantial body of literature, particularly from trials and reviews conducted in China, supports the efficacy of acupoint-based techniques, including acupuncture and acupressure, in managing NVP [64,68,69,70,71]. A systematic review and meta-analysis comparing acupuncture to Western Medicine (WM) found that combining acupuncture with WM may enhance treatment effectiveness for NVP compared to WM alone. Additionally, this approach has been demonstrated to be safe [68]. Other reviews indicate that acupressure may have a potentially beneficial effect in treating NVP. However, robust supportive data are currently lacking [69,70]. While some evidence suggests that acustimulation may help alleviate nausea, vomiting, or hyperemesis during pregnancy, the results remain inconclusive [71]. Furthermore, the absence of standardized guidelines and the limited accessibility of acupoint-based interventions hinder their integration into routine clinical practice.

Although our analysis incorporates the latest developments and trials on the efficacy of available treatments for NVP, comparative assessments of these interventions remain challenging, given that most comparisons are indirect and we identified high heterogeneity due to differences in study characteristics, along with low-to-moderate quality of evidence and sparse reporting of adverse effect profiles. This is primarily due to the lack of high-quality, head-to-head comparative trials with large patient populations and to the inadequate assessment of maternal and fetal complications. According to a 2015 Cochrane review, there is insufficient high-quality evidence to definitively endorse any specific intervention for NVP. This does not imply that the interventions studied are ineffective; rather, it reflects a lack of robust evidence to support the superiority of any single approach [4]. A previous network meta-analysis concluded that current evidence strongly supports the therapeutic benefits of ginger for treating NVP. While several other interventions also demonstrated promising results, the overall strength of the evidence remains very low; thus, the results of this analysis should be interpreted with considerable caution [63]. Our results align with previous studies in this area, as we also observed low-to-moderate quality of evidence and high heterogeneity in study design and population characteristics, even after including the latest studies with stricter inclusion criteria and rigorous analytical methods. Ranking of treatments was also challenging, due to the dominance of indirect comparisons and the lack of safety data reporting.

Standardized symptom severity scores and outcome assessments are critical for advancing research and clinical practice in the management of NVP [4]. Consistent, validated tools, such as the PUQE scale, VAS, and NVP-specific quality-of-life measures, enable reliable comparison of interventions across studies, reducing heterogeneity and improving the robustness of meta-analyses [72,73]. Without standardization, variations in outcome definitions and measurement methods can lead to inconsistent findings, complicating efforts to establish evidence-based guidelines. Additionally, uniform assessment tools facilitate better clinical decision-making by providing clear, comparable benchmarks for symptom severity, treatment efficacy, and patient response. Implementing these standardized measures in future research will enhance data quality and support the development of more precise, patient-centered treatment protocols for NVP.

### 4.3. Strengths and Limitations

This study is strengthened by its strict adherence to internationally recognized guidelines, including PRISMA 2020 [11] and the Cochrane Handbook [12], which ensures methodological rigor and transparency throughout the research process. Furthermore, our analysis includes an extensive range of interventions, spanning pharmacological treatments like doxylamine–pyridoxine and metoclopramide, as well as non-pharmacological approaches such as acupuncture, acupressure, ginger, and dietary supplements, offering a holistic evaluation of available options for managing NVP. Additionally, applying advanced analytical techniques, such as a frequentist network meta-analysis and the CINEMA framework, allows for a comprehensive and nuanced comparison of pharmacological and non-pharmacological interventions, providing clinicians with a robust evidence base for decision-making.

A primary limitation of this study is the considerable heterogeneity observed among the included trials, which arises from differences in participant demographics, intervention protocols, and outcome definitions, thereby complicating the interpretation of comparative effectiveness. While we observed some variability in baseline characteristics or study design, our qualitative assessment of effect modifiers indicated that the included studies were broadly comparable in terms of population characteristics and intervention protocols. This supports the assumption of transitivity for our network meta-analysis. However, we acknowledge that unmeasured or unreported effect modifiers may introduce residual heterogeneity, and we urge cautious interpretation of the findings in light of this potential limitation. The limited reporting of safety outcomes in the primary studies also restricts the ability to conduct a thorough meta-analysis of adverse effects, particularly for pharmacological treatments, where long-term maternal and fetal safety remain a critical concern. Furthermore, the relatively small number of RCTs and participants included in the analysis may limit the generalizability of the findings, especially across diverse populations and clinical settings. Also, the subjective nature of specific outcome measures, such as quality-of-life assessments, introduces potential bias. At the same time, the lack of standardized protocols for non-pharmacological interventions (e.g., acupuncture, ginger dosage) poses challenges for consistent clinical implementation. Finally, the assumption of comparability across scales may introduce residual heterogeneity, and that linear rescaling may not fully account for differences in scale structure, item weighting, or responsiveness. These limitations should be considered when interpreting the pooled estimates.

### 4.4. Implications for Practice and Research

**Figure 5:**
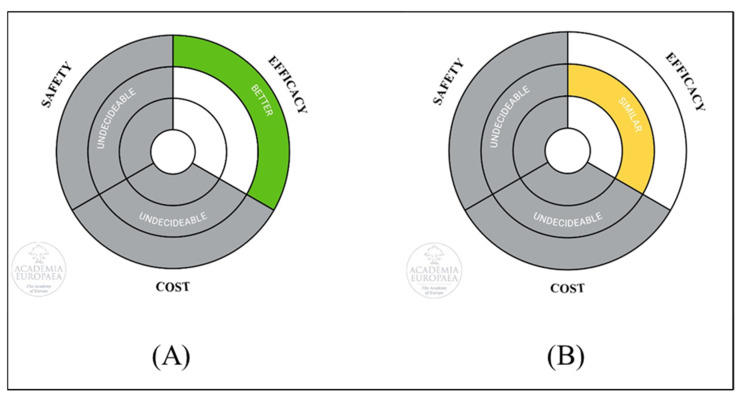
Academia Europaea’s Ring Model for visualizing research implications in publication. (**A**) Comparison of vitamin B6, metoclopramide, dimenhydrinate, ginger, quince, essential oils, acupuncture combined with doxylamine–pyridoxine, acupuncture, and P6 acupressure versus placebo. (**B**) Comparison of other acupressure techniques and doxylamine–pyridoxine versus placebo.

Implementing scientific findings in practice is crucial [10,74]. This review supports the effectiveness of vitamin B6, metoclopramide, dimenhydrinate, ginger, specific dietary supplements, acupuncture, and P6 acupressure in managing NVP, while indicating low-to-moderate quality of evidence. While other acupressure techniques and doxylamine–pyridoxine alone showed clinically relevant improvements, these improvements did not reach statistical significance compared with placebo. Furthermore, data on their long-term safety and cost-effectiveness remain limited (see Figure 5); thus, careful monitoring for adverse effects is warranted [75]. While these interventions demonstrate clinical benefits, their application should be tailored to individual patient needs, considering symptom severity and preferences. However, due to high heterogeneity among studies and limited safety data, especially for pharmacological treatments, clinicians should remain cautious and prioritize shared decision-making. Standardized protocols and additional safety evaluations are necessary to enhance confidence in these recommendations.

Future research should focus on large-scale, high-quality RCTs with standardized outcomes to address the heterogeneity and evidence gaps identified in this review. Prioritizing long-term safety assessments for pharmacological interventions and establishing consistent protocols for non-pharmacological approaches, such as ginger and acupuncture, will improve clinical guidance. Additionally, incorporating cost-effectiveness analyses and patient-centered outcomes can enhance the practical relevance of findings, ultimately informing more robust treatment guidelines for NVP. Careful assessment of maternal and fetal adverse effects is necessary in future studies examining treatments for NVP.

## 5. Conclusions

Our results demonstrate that specific pharmacological treatments (vitamin B6, metoclopramide, and dimenhydrinate) and non-pharmacological interventions (dietary supplements and acupoint-based techniques) are effective in reducing nausea and vomiting during pregnancy. The lack of available safety data warrants careful assessment of potential adverse effects and the development of carefully tailored treatment plans.

## Figures and Tables

**Table 1:** League table of efficacy analysis.

V1	V2	V3	V4	V5	V6	V7	V8	V9	V10	V11	V12	V13	V14	V15	V16	V17
quince	.	.	.	.	.	−1.73 [−2.97; −0.50]	.	.	.	.	.	.	.	.	.	.
−0.57 [−2.27; 1.14]	B6 and pomegranate and mint	.	.	.	.	−1.17 [−2.34; 0.01]	.	.	.	.	.	.	.	.	.	.
−1.39 [−2.78; 0.00]	−0.82 [−2.16; 0.52]	acupressure P6	.	.	.	−0.37 [−1.23; 0.49]	.	−0.68 [−1.98; 0.62]	.	.	.	.	−1.50 [−2.25; −0.76]	.	.	−2.49 [−3.42; −1.55]
−1.42 [−2.93; 0.09]	−0.85 [−2.32; 0.61]	−0.03 [−1.11; 1.05]	dimenhydrinate	.	.	−0.31 [−1.18; 0.56]	.	.	.	.	.	.	.	.	.	.
−1.44 [−3.13; 0.24]	−0.88 [−2.52; 0.77]	−0.05 [−1.25; 1.14]	−0.02 [−1.46; 1.42]	acupuncture doxylamine–pyridoxine	.	.	.	.	.	−0.92 [−1.92; 0.09]	−0.75 [−1.77; 0.27]	.	.	−1.92 [−2.95; −0.89]	.	.
−1.74 [−3.07; −0.41]	−1.17 [−2.45; 0.11]	−0.35 [−1.02; 0.33]	−0.32 [−1.32; 0.68]	−0.30 [−1.39; 0.80]	ginger	−0.17 [−0.73; 0.38]	0.09 [−0.83; 1.01]	.	.	−0.21 [−1.47; 1.05]	.	−0.85 [−1.74; 0.03]	−1.15 [−1.62; −0.69]	.	.	.
−1.73 [−2.97; −0.50]	−1.17 [−2.34; 0.01]	−0.34 [−0.98; 0.30]	−0.31 [−1.18; 0.56]	−0.29 [−1.44; 0.86]	0.01 [−0.49; 0.50]	B6	.	.	.	.	.	.	−1.90 [−2.70; −1.11]	.	.	.
−1.80 [−3.35; −0.24]	−1.23 [−2.74; 0.28]	−0.41 [−1.42; 0.61]	−0.38 [−1.66; 0.91]	−0.35 [−1.68; 0.97]	−0.06 [−0.90; 0.78]	−0.06 [−1.00; 0.88]	metoclopramide	.	.	.	.	.	−0.95 [−1.89; −0.01]	.	.	.
−2.07 [−3.98; −0.17]	−1.50 [−3.37; 0.37]	−0.68 [−1.98; 0.62]	−0.65 [−2.34; 1.04]	−0.63 [−2.40; 1.14]	−0.33 [−1.80; 1.14]	−0.34 [−1.79; 1.11]	−0.27 [−1.93; 1.38]	acupressure KID21	.	.	.	.	.	.	.	.
−2.14 [−3.62; −0.66]	−1.57 [−3.00; −0.14]	−0.75 [−1.61; 0.12]	−0.72 [−1.91; 0.48]	−0.69 [−1.90; 0.52]	−0.40 [−1.15; 0.35]	−0.40 [−1.22; 0.42]	−0.34 [−1.39; 0.71]	−0.07 [−1.63; 1.50]	essential oil	.	.	.	−0.78 [−1.40; −0.16]	.	.	.
−2.28 [−3.76; −0.81]	−1.72 [−3.15; −0.29]	−0.89 [−1.78; −0.01]	−0.86 [−2.05; 0.33]	−0.84 [−1.79; 0.11]	−0.55 [−1.26; 0.17]	−0.55 [−1.36; 0.26]	−0.49 [−1.54; 0.56]	−0.21 [−1.79; 1.36]	−0.15 [−1.05; 0.76]	doxylamine–pyridoxine	0.17 [−0.83; 1.16]	.	−0.58 [−1.53; 0.36]	−1.00 [−2.00; 0.00]	.	.
−2.45 [−3.95; −0.94]	−1.88 [−3.34; −0.42]	−1.06 [−1.98; −0.14]	−1.03 [−2.25; 0.20]	−1.01 [−1.92; −0.09]	−0.71 [−1.50; 0.08]	−0.71 [−1.58; 0.15]	−0.65 [−1.74; 0.43]	−0.38 [−1.97; 1.22]	−0.31 [−1.24; 0.62]	−0.16 [−0.89; 0.56]	acupuncture	.	−0.35 [−1.21; 0.50]	−0.77 [−1.46; −0.07]	−0.66 [−1.63; 0.30]	.
−2.59 [−4.12; −1.06]	−2.03 [−3.51; −0.54]	−1.20 [−2.19; −0.22]	−1.17 [−2.43; 0.08]	−1.15 [−2.45; 0.15]	−0.85 [−1.65; −0.05]	−0.86 [−1.76; 0.05]	−0.80 [−1.92; 0.33]	−0.52 [−2.15; 1.11]	−0.46 [−1.47; 0.56]	−0.31 [−1.32; 0.71]	−0.14 [−1.20; 0.91]	acupressure other	−0.32 [−1.22; 0.57]	.	.	.
−2.91 [−4.26; −1.57]	−2.35 [−3.64; −1.05]	−1.52 [−2.13; −0.91]	−1.49 [−2.52; −0.47]	−1.47 [−2.51; −0.43]	−1.18 [−1.60; −0.75]	−1.18 [−1.72; −0.65]	−1.12 [−1.96; −0.27]	−0.84 [−2.28; 0.59]	−0.78 [−1.40; −0.16]	−0.63 [−1.29; 0.03]	−0.47 [−1.16; 0.23]	−0.32 [−1.13; 0.48]	placebo	.	.	.
−3.18 [−4.78; −1.58]	−2.61 [−4.17; −1.05]	−1.79 [−2.86; −0.72]	−1.76 [−3.10; −0.42]	−1.73 [−2.69; −0.78]	−1.44 [−2.39; −0.49]	−1.44 [−2.46; −0.43]	−1.38 [−2.59; −0.17]	−1.11 [−2.79; 0.58]	−1.04 [−2.12; 0.04]	−0.89 [−1.72; −0.06]	−0.73 [−1.41; −0.05]	−0.59 [−1.77; 0.60]	−0.26 [−1.15; 0.62]	sham acupuncture	−0.25 [−1.25; 0.75]	.
−3.26 [−5.00; −1.52]	−2.69 [−4.39; −0.99]	−1.87 [−3.13; −0.60]	−1.84 [−3.34; −0.33]	−1.81 [−3.04; −0.59]	−1.52 [−2.69; −0.35]	−1.52 [−2.75; −0.30]	−1.46 [−2.85; −0.07]	−1.19 [−3.00; 0.63]	−1.12 [−2.40; 0.15]	−0.97 [−2.08; 0.13]	−0.81 [−1.72; 0.10]	−0.66 [−2.03; 0.70]	−0.34 [−1.46; 0.77]	−0.08 [−1.01; 0.85]	no intervention	.
−3.88 [−5.55; −2.20]	−3.31 [−4.95; −1.67]	−2.49 [−3.42; −1.55]	−2.45 [−3.89; −1.02]	−2.43 [−3.95; −0.91]	−2.14 [−3.29; −0.98]	−2.14 [−3.28; −1.00]	−2.08 [−3.46; −0.69]	−1.81 [−3.41; −0.20]	−1.74 [−3.02; −0.46]	−1.59 [−2.88; −0.30]	−1.43 [−2.74; −0.11]	−1.28 [−2.64; 0.08]	−0.96 [−2.08; 0.16]	−0.70 [−2.12; 0.72]	−0.62 [−2.19; 0.96]	sham acupressure

## Data Availability

The datasets used in this study are available in the full-text articles and Supplementary Materials included in this study.

## Supplementary Materials

The following supporting information can be downloaded at: https://www.mdpi.com/article/10.3390/nu18081293/s1, Supplementary Table S1. PRISMA 2020 checklist; Supplementary S1. Detailed search terms; Supplementary Table S2. Basic characteristics of included studies; Figure S1. Results of risk-of-bias analysis; Figure S2. Results of consistency analysis; Supplementary Table S3. Occurrence of adverse events in included studies; Figure S3. Funnel-plot of efficacy analysis; Supplementary Table S4. Results of CiNeMa analysis. Figure S4: Network plot of efficacy analysis excluding single-study nodes and studies containing digitized outcomes. Figure S5: P-scores of interventions, efficacy analysis excluding single-study nodes and studies containing digitized outcomes. Figure S6: Forest plot with placebo reference, mean difference of NVP symptoms score reduction; efficacy analysis excluding single-study nodes and studies containing digitized outcomes. Figure S7: P-scores of interventions, SMD-based efficacy analysis. Figure S8: Forest plot with placebo reference, mean difference of NVP symptoms score reduction, SMD-based efficacy analysis.
