# Supplementary material for: Comparative Effectiveness of Pharmacological and Non-Pharmacological Interventions for Nausea and Vomiting in Pregnancy: A Systematic Review and Network Meta-Analysis
Source: Nutrients. 2026 Apr 20;18(8):1293. doi: 10.3390/nu18081293 (PMC13118981; doi:10.3390/nu18081293)
Supplement: Supplementary file 1 [file nutrients-18-01293-s001.zip › nutrients-4171759-supplementary.docx]

**SUPPLEMENTARY MATERIALS**

**Title**

Comparative Effectiveness of Pharmacological and Non-pharmacological Interventions for Nausea and Vomiting in Pregnancy: A Systematic Review and Network Meta-Analysis

**Authors**

Lőrinc Frivaldszky^1,2,3^, Mahmoud Obeidat^1^, Péter Hegyi^1,4,5^, Csongor Kárpáti^1^, Zoltán Kobza^1^, Nándor Ács^1,3^, Ferenc Bánhidy^1,3^, Gergely Agócs^1^, Boglárka Szentes^1^, Márton Keszthelyi^1,3^

**Affiliations**

1. Centre for Translational Medicine, Semmelweis University, Baross utca 22, Budapest, H-1085, Hungary
2. MRE Bethesda Children’s Hospital, Bethesda utca 3, Budapest, H-1146, Hungary
3. Department of Obstetrics and Gynecology, Semmelweis University, Üllői út 78/A, Budapest, H-1082, Hungary
4. Institute for Translational Medicine, Medical School, University of Pécs, Szigeti út 12, Pécs, H-7624, Hungary
5. Institute of Pancreatic Diseases, Semmelweis University, Tömő utca 25-29, Budapest, H-1083, Hungary

**Corresponding author**

Márton Keszthelyi MD, PhD

Postal address: H-1082 Budapest, Üllői út 78/A., Hungary

Tel.: +36 1 459 1500

Fax: +36 1 459 1500

E-mail address: [keszthelyi.marton@semmelweis.hu](mailto:keszthelyi.marton@semmelweis.hu)

**TABLE OF CONTENTS**

**Supplementary Table S1**. PRISMA 2020 checklist

**Supplementary S1**. Detailed search terms

**Supplementary Table S2**. Basic characteristics of included studies

**Figure S1**. Results of risk of bias analysis

**Figure S2**. Results of consistency analysis

**Supplementary Table S3**. Occurrence of adverse events in included studies

**Figure S3**. Funnel-plot of efficacy analysis

**Supplementary Table S4**. Results of CiNeMa analysis

**Figure S4:** Network plot of efficacy analysis excluding single-study nodes and studies containing digitized outcomes

**Figure S5:** P-scores of interventions, efficacy analysis excluding single-study nodes and studies containing digitized outcomes

**Figure S6:** Forest plot with placebo reference, mean difference of NVP symptoms score reduction; efficacy analysis excluding single-study nodes and studies containing digitized outcomes

**Figure S7:** P-scores of interventions, SMD-based efficacy analysis

**Figure S8:** Forest plot with placebo reference, mean difference of NVP symptoms score reduction, SMD-based efficacy analysis

**Table S1.** PRISMA 2020 checklist

| **Section and Topic** | **Item #** | **Checklist item** | **Location where item is reported** |
| --- | --- | --- | --- |
| **TITLE** | | |  |
| Title | 1 | Identify the report as a systematic review. | 1 |
| **ABSTRACT** | | |  |
| Abstract | 2 | See the PRISMA 2020 for Abstracts checklist. | 1-2 |
| **INTRODUCTION** | | |  |
| Rationale | 3 | Describe the rationale for the review in the context of existing knowledge. | 2 |
| Objectives | 4 | Provide an explicit statement of the objective(s) or question(s) the review addresses. | 2 |
| **METHODS** | | |  |
| Eligibility criteria | 5 | Specify the inclusion and exclusion criteria for the review and how studies were grouped for the syntheses. | 3 |
| Information sources | 6 | Specify all databases, registers, websites, organisations, reference lists and other sources searched or consulted to identify studies. Specify the date when each source was last searched or consulted. | 3 |
| Search strategy | 7 | Present the full search strategies for all databases, registers and websites, including any filters and limits used. | Supplementary |
| Selection process | 8 | Specify the methods used to decide whether a study met the inclusion criteria of the review, including how many reviewers screened each record and each report retrieved, whether they worked independently, and if applicable, details of automation tools used in the process. | 3 |
| Data collection process | 9 | Specify the methods used to collect data from reports, including how many reviewers collected data from each report, whether they worked independently, any processes for obtaining or confirming data from study investigators, and if applicable, details of automation tools used in the process. | 3 |
| Data items | 10a | List and define all outcomes for which data were sought. Specify whether all results that were compatible with each outcome domain in each study were sought (e.g. for all measures, time points, analyses), and if not, the methods used to decide which results to collect. | 3 |
|  | 10b | List and define all other variables for which data were sought (e.g. participant and intervention characteristics, funding sources). Describe any assumptions made about any missing or unclear information. | 3 |
| Study risk of bias assessment | 11 | Specify the methods used to assess risk of bias in the included studies, including details of the tool(s) used, how many reviewers assessed each study and whether they worked independently, and if applicable, details of automation tools used in the process. | 3 |
| Effect measures | 12 | Specify for each outcome the effect measure(s) (e.g. risk ratio, mean difference) used in the synthesis or presentation of results. | 4 |
| Synthesis methods | 13a | Describe the processes used to decide which studies were eligible for each synthesis (e.g. tabulating the study intervention characteristics and comparing against the planned groups for each synthesis (item #5)). | 4 |
|  | 13b | Describe any methods required to prepare the data for presentation or synthesis, such as handling of missing summary statistics, or data conversions. | 4 |
|  | 13c | Describe any methods used to tabulate or visually display results of individual studies and syntheses. | 4 |
|  | 13d | Describe any methods used to synthesize results and provide a rationale for the choice(s). If meta-analysis was performed, describe the model(s), method(s) to identify the presence and extent of statistical heterogeneity, and software package(s) used. | 4 |
|  | 13e | Describe any methods used to explore possible causes of heterogeneity among study results (e.g. subgroup analysis, meta-regression). | 4 |
|  | 13f | Describe any sensitivity analyses conducted to assess robustness of the synthesized results. | 4 |
| Reporting bias assessment | 14 | Describe any methods used to assess risk of bias due to missing results in a synthesis (arising from reporting biases). | 4 |
| Certainty assessment | 15 | Describe any methods used to assess certainty (or confidence) in the body of evidence for an outcome. | 4 |
| **RESULTS** | | |  |
| Study selection | 16a | Describe the results of the search and selection process, from the number of records identified in the search to the number of studies included in the review, ideally using a flow diagram. | 4 |
|  | 16b | Cite studies that might appear to meet the inclusion criteria, but which were excluded, and explain why they were excluded. | 4 |
| Study characteristics | 17 | Cite each included study and present its characteristics. | 5, Supplementary |
| Risk of bias in studies | 18 | Present assessments of risk of bias for each included study. | 5, Supplementary |
| Results of individual studies | 19 | For all outcomes, present, for each study: (a) summary statistics for each group (where appropriate) and (b) an effect estimate and its precision (e.g. confidence/credible interval), ideally using structured tables or plots. | Supplementary |
| Results of syntheses | 20a | For each synthesis, briefly summarise the characteristics and risk of bias among contributing studies. | 6-7 |
|  | 20b | Present results of all statistical syntheses conducted. If meta-analysis was done, present for each the summary estimate and its precision (e.g. confidence/credible interval) and measures of statistical heterogeneity. If comparing groups, describe the direction of the effect. | 6-7 |
|  | 20c | Present results of all investigations of possible causes of heterogeneity among study results. | 7-8 |
|  | 20d | Present results of all sensitivity analyses conducted to assess the robustness of the synthesized results. | 7-8 |
| Reporting biases | 21 | Present assessments of risk of bias due to missing results (arising from reporting biases) for each synthesis assessed. | 5 |
| Certainty of evidence | 22 | Present assessments of certainty (or confidence) in the body of evidence for each outcome assessed. | 8 |
| **DISCUSSION** | | |  |
| Discussion | 23a | Provide a general interpretation of the results in the context of other evidence. | 11-12 |
|  | 23b | Discuss any limitations of the evidence included in the review. | 12-13 |
|  | 23c | Discuss any limitations of the review processes used. | 12-13 |
|  | 23d | Discuss implications of the results for practice, policy, and future research. | 13-14 |
| **OTHER INFORMATION** | | |  |
| Registration and protocol | 24a | Provide registration information for the review, including register name and registration number, or state that the review was not registered. | 3 |
|  | 24b | Indicate where the review protocol can be accessed, or state that a protocol was not prepared. | 3 |
|  | 24c | Describe and explain any amendments to information provided at registration or in the protocol. | 3 |
| Support | 25 | Describe sources of financial or non-financial support for the review, and the role of the funders or sponsors in the review. | 14-15 |
| Competing interests | 26 | Declare any competing interests of review authors. | 14-15 |
| Availability of data, code and other materials | 27 | Report which of the following are publicly available and where they can be found: template data collection forms; data extracted from included studies; data used for all analyses; analytic code; any other materials used in the review. | Supplementary |

**Supplementary S1.** Detailed search terms

(nausea OR vomiting OR hyperemesis) AND (pregna* OR gravi*) AND (random* OR RCT)

**Table S2**. Basic characteristics of included studies

| **Author (year)** | **Study site** | **Outcomes** | **Number of analyzed**  **patients** | **Intervention** | **Age** | **BMI** | **Parity** | **Gestational week** |
| --- | --- | --- | --- | --- | --- | --- | --- | --- |
| Smith_2002[1] | Australia | VAS, Rhodes Index of  Nausea and Vomiting, Credibility Rating Scale. | 583 | Duration: 3 weeks  int. 1: acupuncture, 20min  int. 2: PC6 acupuncture-20min  int. 3: sham acupuncture-20min  int. 4: no acupuncture | int. 1: 29 ± 4,7  int. 2: 30 ± 4,8  int. 3: 29,6 ± 4,6  int. 4: 30 ± 5,2 | int. 1: NA  int. 2: NA  int. 3: NA  int. 4: NA | int. 1: NA  int. 2: NA  int. 3: NA  int. 4: NA | int. 1: 8,3 (5-13)  int. 2: 8,3 (4-14)  int. 3: 8 (4-13)  int. 4: 8,4 (5-14) |
| Biswas_2011[2] | India | VAS | 63 | Duration: 3 weeks  Int. 1: ginger 150 mg  Int. 2: doxylamine-pyridoxine 20 mg | Int. 1: 21,7 ± 3,06  Int. 2: 22,7 ± 4,46 | Int. 1: NA  Int. 2: NA | Int. 1: NA  Int. 2: NA | Int. 1: NA  Int. 2: NA |
| Galeshi_2020[3] | Iran | VAS | 82 | Duration: 4 days  Int. 1: acupressure KID21  Int. 2: effect of pressure on the (Neiguan) P6 | Int. 1: 26,5 ± 5,5    Int. 2: 28,86 ± 5,94 | Int. 1: 25,97 ± 5,24  Int. 2: 26,04 ± 6,22 | Int. 1: 0,33 ± 0,55  Int. 2: 0,63 ± 0,7 | Int. 1: 9,48 ± 1,99  Int. 2: 9,58 ± 2,45 |
| Adlan_2017[4] | Malaysia | PUQE score | 120 | Duration: 3 days  Int. 1: Acupressure P6  Int. 2: identical non-stimulating wristband | Int. 1: 29 ± 4,92  Int. 2: 28,4 ± 4,34 | Int. 1: NA  Int. 2: NA | Int. 1: 1 (0-2)  Int. 2: 1 (0-2) | Int. 1: 9,7 ± 2,09  Int. 2: 9,2 ± 2,03 |
| Pasha_2012[5] | Iran | VAS, and severity of vomiting | 60 | Duration: 4 days  Int. 1: mint oil  Int. 2: normal saline | Int. 1: 24,8 ± 3,56  Int. 2: 25,1 ± 4,76 | Int. 1: 24,48 ± 2,99  Int. 2: 25,54 ± 3,81 | Int. 1: NA  Int. 2: NA | Int. 1: 9,07 ± 1,31  Int. 2: 9,73 ± 2,21 |
| Abdolhosseini_2017[6] | Iran | PUQE-24 | 55 | Duration: 1 week  Int. 1: Vitamin B6 20 mg  Int. 2: pomegranate syrup plus vitamin B6 20 mg | Int. 1: 27,5 ± 5,6  Int. 2: 26,1 ± 5,7 | Int. 1: 24,2 ± 4,4  Int. 2: 25,5 ± 3,9 | Int. 1: NA  Int. 2: NA | Int. 1: 9,90 ± 4,40  Int. 2: 9,80 ± 3,10 |
| Saberi_2013[7] | Iran | Rhodes index. | 159 | Duration: 4 days  int. 1: acupressure  int. 2: ginger capsules 250mg  int. 3: placebo | int. 1: 25,68 ± 4,64  int. 2: 26,64 ± 6,18  int. 3: 25,79 ± 3,64 | int. 1: NA  int. 2: NA  int. 3: NA | int. 1: NA  int. 2: NA  int. 3: NA | int. 1: 9,32 ± 2,38  int. 2: 8,78 ± 2,32  int. 3: 9,11 ± 0,18 |

| **Author (year)** | **Study site** | **Outcomes** | **Number of analyzed**  **patients** | **Intervention** | **Age** | **BMI** | **Parity** | **Gestational week** |
| --- | --- | --- | --- | --- | --- | --- | --- | --- |
| Yavari Kia_2014[8] | Iran | PUQE-24 | 100 | Duration: 4 days  Int. 1: lemon oil  Int. 2: placebo | Int. 1: 26,2 ± 5,58  Int. 2: 25,76 ± 5,8 | Int. 1: NA  Int. 2: NA | Int. 1: NA  Int. 2: NA | Int. 1: 10,32 ± 2,45  Int. 2: 10,98 ± 2,76 |
| Firouzbakht_2014[9] | Iran | VAS | 120 | Duration: 4 days  int. 1: B6 40mg  int. 2: placebo  int. 3: ginger capsule 250mg | int. 1: 24,43 ± 3,70  int. 2: 25,39 ± 5,24  int. 3: 24,90 ± 5,50 | int. 1: NA  int. 2: NA  int. 3: NA | int. 1: NA  int. 2: NA  int. 3: NA | int. 1: 8,90 ± 2,90  int. 2: 9,10 ± 3,60  int. 3: 9,10 ± 4,60 |
| Mohammadbeigi_2011[10] | Iran | Rhodes Index | 102 | Duration: 5 days  int. 1: metoclopramide 10mg  int. 2: ginger 200mg  int. 3: flour 200mg | int. 1: 27,88 ± 2,21  int. 2: 26,94 ± 3,94  int. 3: 26,97 ± 4,22 | int. 1: NA  int. 2: NA  int. 3: NA | int. 1: NA  int. 2: NA  int. 3: NA | int. 1: 10,03 ± 1,99  int. 2: 9,50 ± 2,02  int. 3: 10,32 ± 2,25 |
| Moraes Froes_2024[11] | Brazil | RINVR | 58 | Duration: 7 days  Int. 1: auriculotherapy  Int. 2: sham auriculotherapy | Int. 1: 25,00 ± 5,48  Int. 2: 25,00 ± 5,48 | Int. 1: NA  Int. 2: NA | Int. 1: NA  Int. 2: NA | Int. 1: NA  Int. 2: NA |
| Safajou_2020[12] | Iran | PUQE‐24, FSS | 90 | Duration: 4 days  Int. 1: lemon oil + peppermint oil  Int. 2: placebo | Int. 1: 27,15 ± 5,11  Int. 2: 26,13 ± 4,56 | Int. 1: NA  Int. 2: NA | Int. 1: NA  Int. 2: NA | Int. 1: 11,11 ± 2,97  Int. 2: 10,45 ± 3,12 |
| Yilmaz_2023[13] | Turkey | PUQE | 74 | Duration: 7 days  Int. 1: Acupressure P6  Int. 2: Control | Int. 1: 28,7 ± 6,12  Int. 2: 28,84 ± 5,14 | Int. 1: NA  Int. 2: NA | Int. 1: NA  Int. 2: NA | Int. 1: 9,32 ± 2,16  Int. 2: 8,76 ± 2,02 |
| **Author (year)** | **Study site** | **Outcomes** | **Number of analyzed**  **patients** | **Intervention** | **Age** | **BMI** | **Parity** | **Gestational week** |
| Chittumma_2007[14] | Thailand | Rhodes score | 126 | Duration: 4 days  Int. 1: Ginger 650mg  Int. 2: Vitamin B6 25mg | Int. 1: 23,8 ± 5,10  Int. 2: 24,4 ± 5,30 | Int. 1: NA  Int. 2: NA | Int. 1: NA  Int. 2: NA | Int. 1: 12,00 ± 2,00  Int. 2: 11,00 ± 2,00 |
| Kirca_2020[15] | Turkey | NVPI-score | 149 | Duration: 7 days  Int. 1: Acupressure P6  Int. 2: Control | Int. 1: 28 (20-38)  Int. 2: 27 (18-40) | Int. 1: 23,4 (16,6-42,6)  Int. 2: 24,2 (17,4-45,5) | Int. 1: NA  Int. 2: NA | Int. 1: 9 (7-12)  Int. 2: 10 (6-11) |
| Basirat_2009[16] | Iran | VAS, number of vomiting epi-  sodes | 65 | Duration: 4 days  Int. 1: Ginger 500mg  Int. 2: placebo biscuit | Int. 1: NA  Int. 2: NA | Int. 1: NA  Int. 2: NA | Int. 1: NA  Int. 2: NA | Int. 1: NA  Int. 2: NA |
| Negarandeh_2020[17] | Iran | Rhodes Index. | 128 | Duration: 3 days  Int. 1: auriculotherapy  Int. 2: Control | Int. 1: NA  Int. 2: NA | Int. 1: NA  Int. 2: NA | Int. 1: NA  Int. 2: NA | Int. 1: NA  Int. 2: NA |
| Jamigorn_2007[18] | Thailand | Rhodes index. Secondary out-  come measures were weight gain and medication use. | 60 | Duration: 5 days  Int. 1: Acupressure P6  Int. 2: Sham acupressure | Int. 1: 28,20 ± 5,10  Int. 2: 28,10 ± 5,60 | Int. 1: NA  Int. 2: NA | Int. 1: NA  Int. 2: NA | Int. 1: 8,10 ± 1,70  Int. 2: 8,90 ± 3,50 |
| Babaei_2014[19] | Iran | Rhodes score. | 134 | Duration: 7 days  Int. 1: Vitamin B6 50mg  Int. 2: Dimenhydrinate 50mg | Int. 1: 24,80 ± 2,10  Int. 2: 25,40 ± 3,30 | Int. 1: 19,42 ± 3,2  Int. 2: 20,11 ± 1,70 | Int. 1: NA  Int. 2: NA | Int. 1: 10,00 ± 7,00  Int. 2: 11,00 ± 3,00 |

| **Author (year)** | **Study site** | **Outcomes** | **Number of analyzed**  **patients** | **Intervention** | **Age** | **BMI** | **Parity** | **Gestational week** |
| --- | --- | --- | --- | --- | --- | --- | --- | --- |
| Sharifzadeh_2017[20] | Iran | Rhodes questionnaire | 78 | Duration: 4 days  int. 1: Ginger 500mg  int. 2: Ginger 40mg  int. 3: flour 200mg | int. 1: 28,95 ± 0,5  int. 2: 28,03 ± 3,70  int. 3: 29,03 ± 5,20 | int. 1: NA  int. 2: NA  int. 3: NA | int. 1: NA  int. 2: NA  int. 3: NA | int. 1: 10,90 ± 4,6  int. 2: 10,80 ± 4,80  int. 3: 10,90 ± 3,60 |
| Joulaeerad_2018[21] | Iran | PUQE | 56 | Duration: 4 days  Int. 1: Peppermint oil  Int. 2: Placebo | Int. 1: 26,39 ± 4,27  Int. 2: 27,79 ± 3,51 | Int. 1: NA  Int. 2: NA | Int. 1: 0,82 ± 0,67  Int. 2: 0,89 ± 0,73 | Int. 1: 12,40 ± 3,77  Int. 2: 12,10 ± 4,06 |
| Koren_2016[22] | Canada | PUQE score | 234 | Duration: 14 days  Int. 1: doxylamine succinate + pyridoxine hydrochloride 10-10mg  Int. 2: Placebo | Int. 1: NA  Int. 2: NA | Int. 1: NA  Int. 2: NA | Int. 1: NA  Int. 2: NA | Int. 1: NA  Int. 2: NA |
| Wu_2023[23] | China | PUQE score. Secondary outcomes included quality of life, adverse events,  and maternal and perinatal complications. | 352 | Duration: 14 days  int. 1: Active acupuncture + Doxylamin -Pyridoxin 10-10mg  int. 2: sham acupuncture + Doxylamin -Pyridoxin 10-10mg  int. 3: active acupuncture+ placebo  int. 4: sham acupuncture+ placebo | int. 1: 28,80 ± 4,00  int. 2: 28,60 ± 4,50  int. 3: 29,60 ± 4,60  int. 4: 29,20 ± 4,20 | int. 1: 21,80 ± 3,80  int. 2: 21,10 ± 3,00  int. 3: 21,60 ± 3,30  int. 4: 21,10 ± 2,90 | int. 1: NA  int. 2: NA  int. 3: NA  int. 4: NA | int. 1: NA  int. 2: NA  int. 3: NA  int. 4: NA |
| Dehkordi_2017[24] | Iran | PUQE-24 | 76 | Duration: 7 days  Int. 1: Quince syrup  Int. 2: Vitamin B6 20mg | Int. 1: 27,35 ± 5,19  Int. 2: 27,81 ± 5,45 | Int. 1: 24,22 ± 1,30  Int. 2: 24,90 ± 2,02 | Int. 1: NA  Int. 2: NA | Int. 1: NA  Int. 2: NA |

RINVR: Rhodes Index of Nausea, Vomiting, and Retching, FSS: Fatigue Severity Scale, PUQE‐24: 24‐hour Pregnancy Unique Quantization of Emesis, PUQE: Pregnancy-Unique Quantification of Emesis, VAS: visual analog scales, NVPI: Nausea and Vomiting in Pregnancy Instrument, NVPQOL: Nausea and Vomiting in Pregnancy Quality of Life


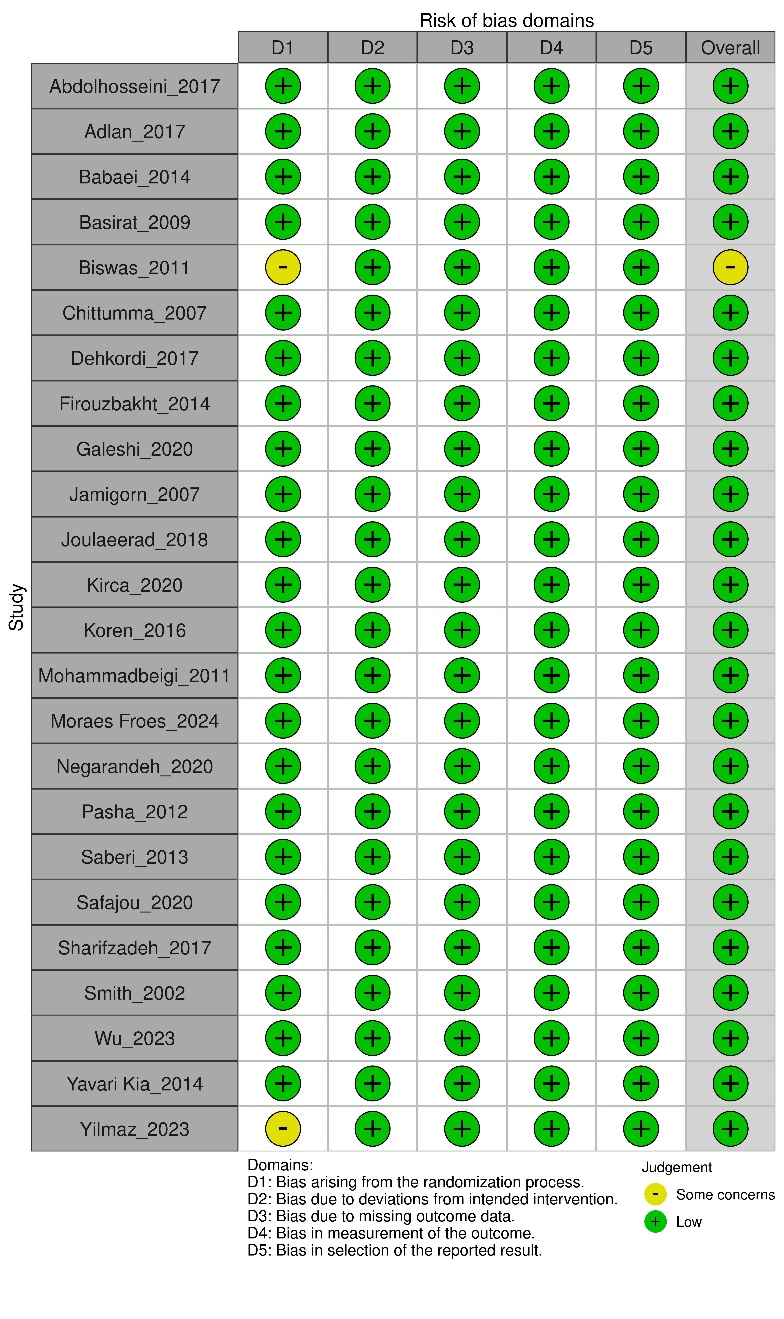


**Figure S1**. Results of risk of bias analysis


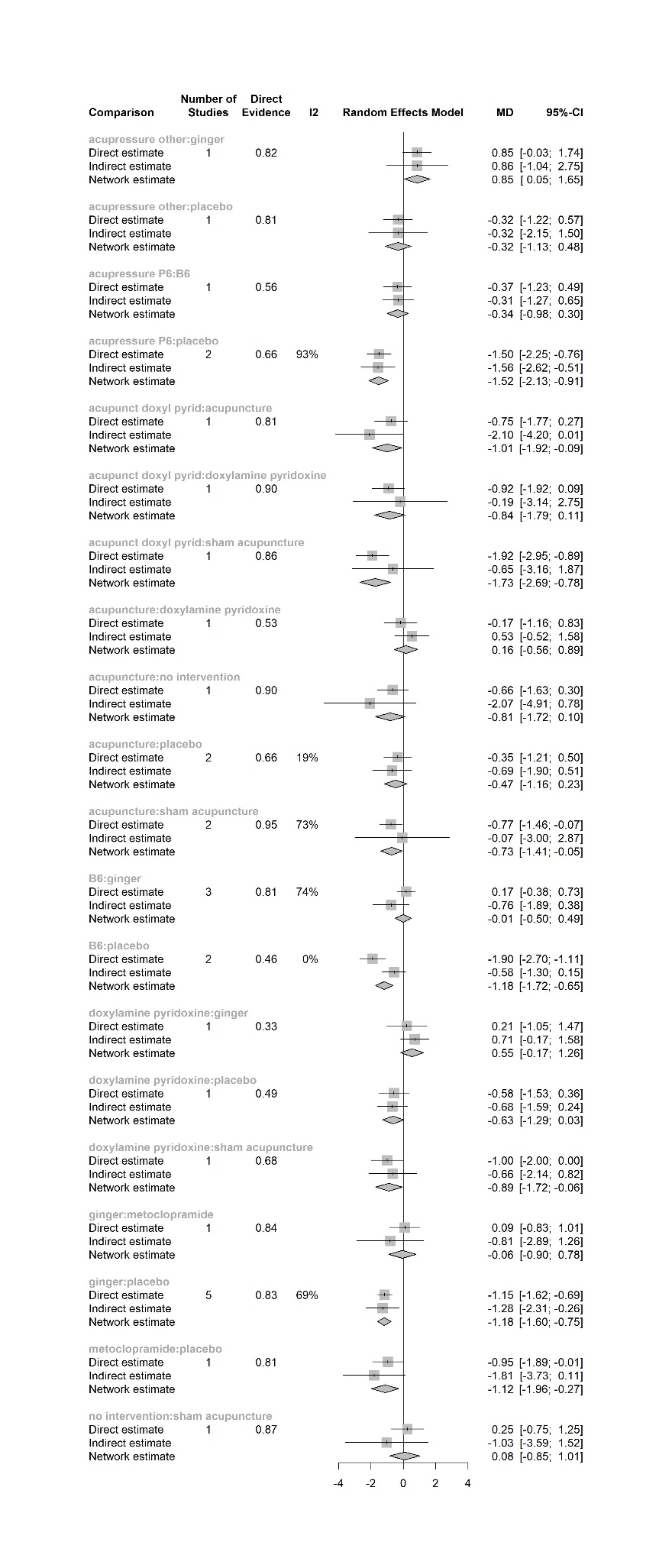


**Figure S2**. Results of consistency analysis

**Table S3**. Occurrence of adverse events in included studies

| Study | Adverse effect, number of patients involved (rate of affected patients in study arm) |
| --- | --- |
| Biswas_2011[2] | Ginger: loose stools 1 (2,94%)  Doxylamine-pyridoxine: hyperacidity 2 (6,89%) |
| Chittumma_2007[14] | Ginger: heartburn 8 (12,1%); sedation 7 (11,1%); arrhythmia 1 (1,6%)  Vitamin B6: heartburn 2 (3,2%); sedation 11 (17,5%); headache 2 (3,1%) |
| Basirat_2009[16] | Ginger: dizziness 1 (3,12%); heartburn 1 (3,2%)  Placebo: N/A |
| Jamigorn_2007[18] | Acupressure: irritation 1 (3,03%)  Vitamin B6: N/A |
| Babaei_2014[19] | Vitamin B6: drowsiness 5 (4,46%)  Dimenhydrinate: drowsiness 36 (52,94%) |
| Joulaeerad_2018[21] | Peppermint oil: headache 2 (3,57%); dizziness 1 (1,79%); shortness of breath 1 (1,79%)  Placebo: headache 1 (1,79%) |


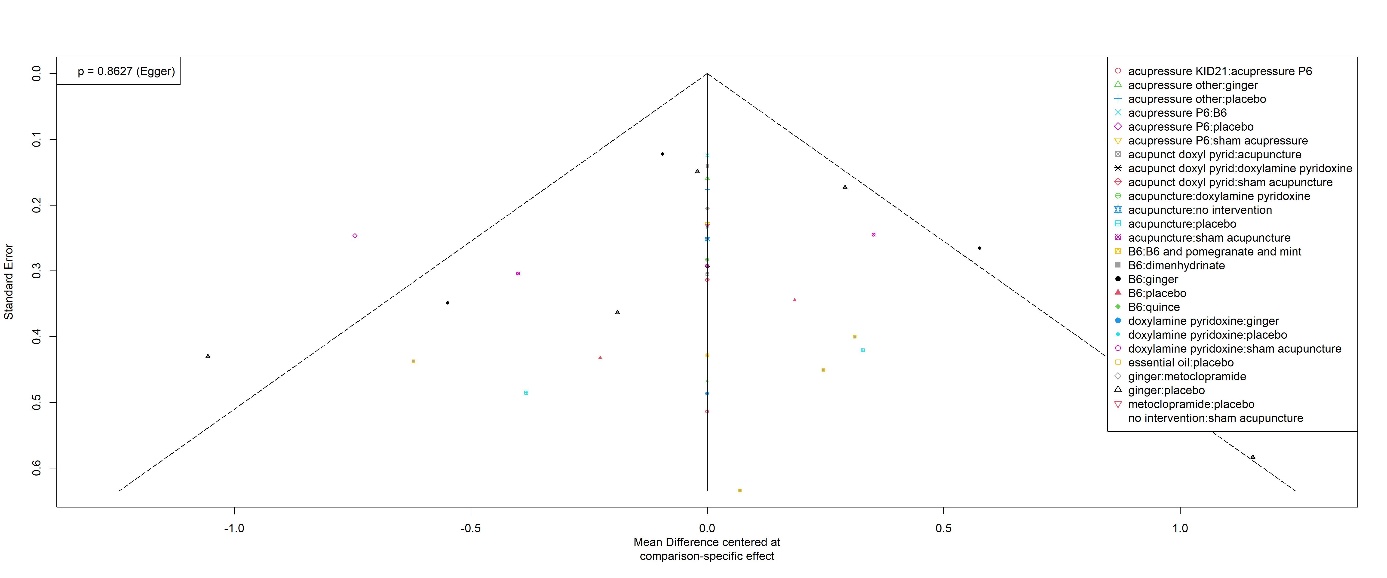


**Figure S3**. Funnel-plot of efficacy analysis

**Table S4**. Results of CiNeMa analysis

| **Comparison** | **Number of studies** | **Within-study bias** | **Reporting bias** | **Indirectness** | **Imprecision** | **Heterogeneity** | **Incoherence** | **Confidence rating** | **Reason(s) for downgrading** |
| --- | --- | --- | --- | --- | --- | --- | --- | --- | --- |
| B6:B6_pomegranate_mint | 1 | No concerns | Some concerns | No concerns | Some concerns | No concerns | No concerns | Low | ["Reporting bias","Imprecision"] |
| acupressure_P6:B6 | 1 | No concerns | Some concerns | No concerns | No concerns | No concerns | No concerns | Moderate | ["Reporting bias"] |
| B6:dimenhydrinate | 1 | No concerns | Some concerns | No concerns | No concerns | Some concerns | No concerns | Low | ["Reporting bias","Heterogeneity"] |
| B6:ginger | 3 | No concerns | Some concerns | No concerns | No concerns | No concerns | No concerns | Moderate | ["Reporting bias"] |
| B6:placebo | 2 | No concerns | Some concerns | No concerns | No concerns | Some concerns | Some concerns | Very low | ["Reporting bias","Heterogeneity","Incoherence"] |
| B6:quince | 1 | No concerns | Some concerns | No concerns | No concerns | Some concerns | No concerns | Low | ["Reporting bias","Heterogeneity"] |
| acupressure_KID21:acupressure_P6 | 1 | No concerns | Some concerns | No concerns | Some concerns | No concerns | No concerns | Low | ["Reporting bias","Imprecision"] |
| acupressure_P6:ginger | 1 | No concerns | Some concerns | No concerns | No concerns | No concerns | No concerns | Moderate | ["Reporting bias"] |
| acupressure_P6:placebo | 3 | No concerns | Some concerns | No concerns | No concerns | Some concerns | No concerns | Low | ["Reporting bias","Heterogeneity"] |
| acupressure_P6:sham_acupressure | 1 | No concerns | Some concerns | No concerns | No concerns | No concerns | No concerns | Moderate | ["Reporting bias"] |
| acupunct_doxyl_pyrid:acupuncture | 1 | No concerns | Some concerns | No concerns | Some concerns | No concerns | No concerns | Low | ["Reporting bias","Imprecision"] |
| acupunct_doxyl_pyrid:doxylamine_pyridoxine | 1 | No concerns | Some concerns | No concerns | Some concerns | No concerns | No concerns | Low | ["Reporting bias","Imprecision"] |
| acupunct_doxyl_pyrid:sham_acupuncture | 1 | No concerns | Some concerns | No concerns | No concerns | Some concerns | No concerns | Low | ["Reporting bias","Heterogeneity"] |
| acupuncture:doxylamine_pyridoxine | 1 | No concerns | Some concerns | No concerns | No concerns | No concerns | No concerns | Moderate | ["Reporting bias"] |
| acupuncture:no_intervention | 1 | No concerns | Some concerns | No concerns | No concerns | Some concerns | No concerns | Low | ["Reporting bias","Heterogeneity"] |
| acupuncture:placebo | 2 | No concerns | Some concerns | No concerns | No concerns | No concerns | No concerns | Moderate | ["Reporting bias"] |
| acupuncture:sham_acupuncture | 2 | No concerns | Some concerns | No concerns | No concerns | Some concerns | No concerns | Low | ["Reporting bias","Heterogeneity"] |
| doxylamine_pyridoxine:ginger | 1 | No concerns | Some concerns | No concerns | No concerns | Some concerns | No concerns | Low | ["Reporting bias","Heterogeneity"] |
| doxylamine_pyridoxine:placebo | 1 | No concerns | Some concerns | No concerns | No concerns | Some concerns | No concerns | Low | ["Reporting bias","Heterogeneity"] |
| doxylamine_pyridoxine:sham_acupuncture | 1 | No concerns | Some concerns | No concerns | No concerns | Some concerns | No concerns | Low | ["Reporting bias","Heterogeneity"] |
| essential_oil:placebo | 4 | No concerns | Some concerns | No concerns | No concerns | Some concerns | No concerns | Low | ["Reporting bias","Heterogeneity"] |
| ginger:metoclopramide | 1 | No concerns | Some concerns | No concerns | No concerns | No concerns | No concerns | Moderate | ["Reporting bias"] |
| ginger:placebo | 5 | No concerns | Some concerns | No concerns | No concerns | Some concerns | No concerns | Low | ["Reporting bias","Heterogeneity"] |
| metoclopramide:placebo | 1 | No concerns | Some concerns | No concerns | No concerns | Some concerns | No concerns | Low | ["Reporting bias","Heterogeneity"] |
| no_intervention:sham_acupuncture | 1 | No concerns | Some concerns | No concerns | No concerns | No concerns | No concerns | Moderate | ["Reporting bias"] |
| acupressure_KID21:B6 | 0 | No concerns | Some concerns | No concerns | Some concerns | No concerns | No concerns | Low | ["Reporting bias","Imprecision"] |
| acupunct_doxyl_pyrid:B6 | 0 | No concerns | Some concerns | No concerns | No concerns | Some concerns | No concerns | Low | ["Reporting bias","Heterogeneity"] |
| acupuncture:B6 | 0 | No concerns | Some concerns | No concerns | No concerns | Some concerns | No concerns | Low | ["Reporting bias","Heterogeneity"] |
| B6:doxylamine_pyridoxine | 0 | No concerns | Some concerns | No concerns | No concerns | Some concerns | No concerns | Low | ["Reporting bias","Heterogeneity"] |
| B6:essential_oil | 0 | No concerns | Some concerns | No concerns | No concerns | No concerns | No concerns | Moderate | ["Reporting bias"] |
| B6:metoclopramide | 0 | No concerns | Some concerns | No concerns | No concerns | No concerns | No concerns | Moderate | ["Reporting bias"] |
| B6:no_intervention | 0 | No concerns | Some concerns | No concerns | Some concerns | No concerns | No concerns | Low | ["Reporting bias","Imprecision"] |
| B6:sham_acupressure | 0 | No concerns | Some concerns | No concerns | No concerns | No concerns | No concerns | Moderate | ["Reporting bias"] |
| B6:sham_acupuncture | 0 | No concerns | Some concerns | No concerns | No concerns | Some concerns | No concerns | Low | ["Reporting bias","Heterogeneity"] |
| acupressure_KID21:B6_pomegranate_mint | 0 | No concerns | Some concerns | No concerns | Some concerns | No concerns | No concerns | Low | ["Reporting bias","Imprecision"] |
| acupressure_P6:B6_pomegranate_mint | 0 | No concerns | Some concerns | No concerns | Some concerns | No concerns | No concerns | Low | ["Reporting bias","Imprecision"] |
| acupunct_doxyl_pyrid:B6_pomegranate_mint | 0 | No concerns | Some concerns | No concerns | Some concerns | No concerns | No concerns | Low | ["Reporting bias","Imprecision"] |
| acupuncture:B6_pomegranate_mint | 0 | No concerns | Some concerns | No concerns | No concerns | Some concerns | No concerns | Low | ["Reporting bias","Heterogeneity"] |
| B6_pomegranate_mint:dimenhydrinate | 0 | No concerns | Some concerns | No concerns | Some concerns | No concerns | No concerns | Low | ["Reporting bias","Imprecision"] |
| B6_pomegranate_mint:doxylamine_pyridoxine | 0 | No concerns | Some concerns | No concerns | Some concerns | No concerns | No concerns | Low | ["Reporting bias","Imprecision"] |
| B6_pomegranate_mint:essential_oil | 0 | No concerns | Some concerns | No concerns | Some concerns | No concerns | No concerns | Low | ["Reporting bias","Imprecision"] |
| B6_pomegranate_mint:ginger | 0 | No concerns | Some concerns | No concerns | Some concerns | No concerns | No concerns | Low | ["Reporting bias","Imprecision"] |
| B6_pomegranate_mint:metoclopramide | 0 | No concerns | Some concerns | No concerns | Some concerns | No concerns | No concerns | Low | ["Reporting bias","Imprecision"] |
| B6_pomegranate_mint:no_intervention | 0 | No concerns | Some concerns | No concerns | No concerns | No concerns | No concerns | Moderate | ["Reporting bias"] |
| B6_pomegranate_mint:placebo | 0 | No concerns | Some concerns | No concerns | No concerns | No concerns | No concerns | Moderate | ["Reporting bias"] |
| B6_pomegranate_mint:quince | 0 | No concerns | Some concerns | No concerns | Some concerns | No concerns | No concerns | Low | ["Reporting bias","Imprecision"] |
| B6_pomegranate_mint:sham_acupressure | 0 | No concerns | Some concerns | No concerns | No concerns | No concerns | No concerns | Moderate | ["Reporting bias"] |
| B6_pomegranate_mint:sham_acupuncture | 0 | No concerns | Some concerns | No concerns | No concerns | No concerns | No concerns | Moderate | ["Reporting bias"] |
| acupressure_KID21:acupunct_doxyl_pyrid | 0 | No concerns | Some concerns | No concerns | Some concerns | No concerns | No concerns | Low | ["Reporting bias","Imprecision"] |
| acupressure_KID21:acupuncture | 0 | No concerns | Some concerns | No concerns | No concerns | Major concerns | No concerns | Very low | ["Reporting bias","Heterogeneity"] |
| acupressure_KID21:dimenhydrinate | 0 | No concerns | Some concerns | No concerns | Some concerns | No concerns | No concerns | Low | ["Reporting bias","Imprecision"] |
| acupressure_KID21:doxylamine_pyridoxine | 0 | No concerns | Some concerns | No concerns | Some concerns | Some concerns | No concerns | Very low | ["Reporting bias","Imprecision","Heterogeneity"] |
| acupressure_KID21:essential_oil | 0 | No concerns | Some concerns | No concerns | Some concerns | No concerns | No concerns | Low | ["Reporting bias","Imprecision"] |
| acupressure_KID21:ginger | 0 | No concerns | Some concerns | No concerns | Some concerns | No concerns | No concerns | Low | ["Reporting bias","Imprecision"] |
| acupressure_KID21:metoclopramide | 0 | No concerns | Some concerns | No concerns | Some concerns | No concerns | No concerns | Low | ["Reporting bias","Imprecision"] |
| acupressure_KID21:no_intervention | 0 | No concerns | Some concerns | No concerns | Some concerns | No concerns | No concerns | Low | ["Reporting bias","Imprecision"] |
| acupressure_KID21:placebo | 0 | No concerns | Some concerns | No concerns | Some concerns | No concerns | No concerns | Low | ["Reporting bias","Imprecision"] |
| acupressure_KID21:quince | 0 | No concerns | Some concerns | No concerns | No concerns | Some concerns | No concerns | Low | ["Reporting bias","Heterogeneity"] |
| acupressure_KID21:sham_acupressure | 0 | No concerns | Some concerns | No concerns | Some concerns | No concerns | No concerns | Low | ["Reporting bias","Imprecision"] |
| acupressure_KID21:sham_acupuncture | 0 | No concerns | Some concerns | No concerns | Some concerns | No concerns | No concerns | Low | ["Reporting bias","Imprecision"] |
| acupressure_P6:acupunct_doxyl_pyrid | 0 | No concerns | Some concerns | No concerns | No concerns | Some concerns | No concerns | Low | ["Reporting bias","Heterogeneity"] |
| acupressure_P6:acupuncture | 0 | No concerns | Some concerns | No concerns | No concerns | Some concerns | No concerns | Low | ["Reporting bias","Heterogeneity"] |
| acupressure_P6:dimenhydrinate | 0 | No concerns | Some concerns | No concerns | No concerns | Some concerns | No concerns | Low | ["Reporting bias","Heterogeneity"] |
| acupressure_P6:doxylamine_pyridoxine | 0 | No concerns | Some concerns | No concerns | No concerns | Some concerns | No concerns | Low | ["Reporting bias","Heterogeneity"] |
| acupressure_P6:essential_oil | 0 | No concerns | Some concerns | No concerns | No concerns | No concerns | No concerns | Moderate | ["Reporting bias"] |
| acupressure_P6:metoclopramide | 0 | No concerns | Some concerns | No concerns | No concerns | No concerns | No concerns | Moderate | ["Reporting bias"] |
| acupressure_P6:no_intervention | 0 | No concerns | Some concerns | No concerns | Some concerns | No concerns | No concerns | Low | ["Reporting bias","Imprecision"] |
| acupressure_P6:quince | 0 | No concerns | Some concerns | No concerns | No concerns | Some concerns | No concerns | Low | ["Reporting bias","Heterogeneity"] |
| acupressure_P6:sham_acupuncture | 0 | No concerns | Some concerns | No concerns | No concerns | Some concerns | No concerns | Low | ["Reporting bias","Heterogeneity"] |
| acupunct_doxyl_pyrid:dimenhydrinate | 0 | No concerns | Some concerns | No concerns | No concerns | Major concerns | No concerns | Very low | ["Reporting bias","Heterogeneity"] |
| acupunct_doxyl_pyrid:essential_oil | 0 | No concerns | Some concerns | No concerns | Some concerns | No concerns | No concerns | Low | ["Reporting bias","Imprecision"] |
| acupunct_doxyl_pyrid:ginger | 0 | No concerns | Some concerns | No concerns | No concerns | Some concerns | No concerns | Low | ["Reporting bias","Heterogeneity"] |
| acupunct_doxyl_pyrid:metoclopramide | 0 | No concerns | Some concerns | No concerns | Some concerns | No concerns | No concerns | Low | ["Reporting bias","Imprecision"] |
| acupunct_doxyl_pyrid:no_intervention | 0 | No concerns | Some concerns | No concerns | No concerns | Some concerns | No concerns | Low | ["Reporting bias","Heterogeneity"] |
| acupunct_doxyl_pyrid:placebo | 0 | No concerns | Some concerns | No concerns | No concerns | Some concerns | No concerns | Low | ["Reporting bias","Heterogeneity"] |
| acupunct_doxyl_pyrid:quince | 0 | No concerns | Some concerns | No concerns | Some concerns | No concerns | No concerns | Low | ["Reporting bias","Imprecision"] |
| acupunct_doxyl_pyrid:sham_acupressure | 0 | No concerns | Some concerns | No concerns | No concerns | No concerns | No concerns | Moderate | ["Reporting bias"] |
| acupuncture:dimenhydrinate | 0 | No concerns | Some concerns | No concerns | Some concerns | No concerns | No concerns | Low | ["Reporting bias","Imprecision"] |
| acupuncture:essential_oil | 0 | No concerns | Some concerns | No concerns | No concerns | Some concerns | No concerns | Low | ["Reporting bias","Heterogeneity"] |
| acupuncture:ginger | 0 | No concerns | Some concerns | No concerns | No concerns | Some concerns | No concerns | Low | ["Reporting bias","Heterogeneity"] |
| acupuncture:metoclopramide | 0 | No concerns | Some concerns | No concerns | Some concerns | No concerns | No concerns | Low | ["Reporting bias","Imprecision"] |
| acupuncture:quince | 0 | No concerns | Some concerns | No concerns | No concerns | No concerns | No concerns | Low | ["Reporting bias"] |
| acupuncture:sham_acupressure | 0 | No concerns | Some concerns | No concerns | No concerns | Some concerns | No concerns | Low | ["Reporting bias","Heterogeneity"] |
| dimenhydrinate:doxylamine_pyridoxine | 0 | No concerns | Some concerns | No concerns | Some concerns | No concerns | No concerns | Low | ["Reporting bias","Imprecision"] |
| dimenhydrinate:essential_oil | 0 | No concerns | Some concerns | No concerns | Some concerns | No concerns | No concerns | Low | ["Reporting bias","Imprecision"] |
| dimenhydrinate:ginger | 0 | No concerns | Some concerns | No concerns | No concerns | Some concerns | No concerns | Low | ["Reporting bias","Heterogeneity"] |
| dimenhydrinate:metoclopramide | 0 | No concerns | Some concerns | No concerns | No concerns | Some concerns | No concerns | Low | ["Reporting bias","Heterogeneity"] |
| dimenhydrinate:no_intervention | 0 | No concerns | Some concerns | No concerns | Some concerns | No concerns | No concerns | Low | ["Reporting bias","Imprecision"] |
| dimenhydrinate:placebo | 0 | No concerns | Some concerns | No concerns | No concerns | Some concerns | No concerns | Low | ["Reporting bias","Heterogeneity"] |
| dimenhydrinate:quince | 0 | No concerns | Some concerns | No concerns | Some concerns | No concerns | No concerns | Low | ["Reporting bias","Imprecision"] |
| dimenhydrinate:sham_acupressure | 0 | No concerns | Some concerns | No concerns | No concerns | No concerns | No concerns | Moderate | ["Reporting bias"] |
| dimenhydrinate:sham_acupuncture | 0 | No concerns | Some concerns | No concerns | Some concerns | No concerns | No concerns | Low | ["Reporting bias","Imprecision"] |
| doxylamine_pyridoxine:essential_oil | 0 | No concerns | Some concerns | No concerns | No concerns | No concerns | No concerns | Moderate | ["Reporting bias"] |
| doxylamine_pyridoxine:metoclopramide | 0 | No concerns | Some concerns | No concerns | No concerns | Some concerns | No concerns | Low | ["Reporting bias","Heterogeneity"] |
| doxylamine_pyridoxine:no_intervention | 0 | No concerns | Some concerns | No concerns | Some concerns | No concerns | No concerns | Low | ["Reporting bias","Imprecision"] |
| doxylamine_pyridoxine:quince | 0 | No concerns | Some concerns | No concerns | No concerns | Some concerns | No concerns | Low | ["Reporting bias","Heterogeneity"] |
| doxylamine_pyridoxine:sham_acupressure | 0 | No concerns | Some concerns | No concerns | No concerns | Some concerns | No concerns | Low | ["Reporting bias","Heterogeneity"] |
| essential_oil:ginger | 0 | No concerns | Some concerns | No concerns | No concerns | Some concerns | No concerns | Low | ["Reporting bias","Heterogeneity"] |
| essential_oil:metoclopramide | 0 | No concerns | Some concerns | No concerns | No concerns | Some concerns | No concerns | Low | ["Reporting bias","Heterogeneity"] |
| essential_oil:no_intervention | 0 | No concerns | Some concerns | No concerns | Some concerns | No concerns | No concerns | Low | ["Reporting bias","Imprecision"] |
| essential_oil:quince | 0 | No concerns | Some concerns | No concerns | No concerns | Some concerns | No concerns | Low | ["Reporting bias","Heterogeneity"] |
| essential_oil:sham_acupressure | 0 | No concerns | Some concerns | No concerns | No concerns | No concerns | No concerns | Moderate | ["Reporting bias"] |
| essential_oil:sham_acupuncture | 0 | No concerns | Some concerns | No concerns | Some concerns | No concerns | No concerns | Low | ["Reporting bias","Imprecision"] |
| ginger:no_intervention | 0 | No concerns | Some concerns | No concerns | No concerns | Some concerns | No concerns | Low | ["Reporting bias","Heterogeneity"] |
| ginger:quince | 0 | No concerns | Some concerns | No concerns | No concerns | Some concerns | No concerns | Low | ["Reporting bias","Heterogeneity"] |
| ginger:sham_acupressure | 0 | No concerns | Some concerns | No concerns | No concerns | No concerns | No concerns | Moderate | ["Reporting bias"] |
| ginger:sham_acupuncture | 0 | No concerns | Some concerns | No concerns | No concerns | Some concerns | No concerns | Low | ["Reporting bias","Heterogeneity"] |
| metoclopramide:no_intervention | 0 | No concerns | Some concerns | No concerns | Some concerns | No concerns | No concerns | Low | ["Reporting bias","Imprecision"] |
| metoclopramide:quince | 0 | No concerns | Some concerns | No concerns | Some concerns | No concerns | No concerns | Low | ["Reporting bias","Imprecision"] |
| metoclopramide:sham_acupressure | 0 | No concerns | Some concerns | No concerns | No concerns | No concerns | No concerns | Moderate | ["Reporting bias"] |
| metoclopramide:sham_acupuncture | 0 | No concerns | Some concerns | No concerns | Some concerns | No concerns | No concerns | Low | ["Reporting bias","Imprecision"] |
| no_intervention:placebo | 0 | No concerns | Some concerns | No concerns | No concerns | Some concerns | No concerns | Low | ["Reporting bias","Heterogeneity"] |
| no_intervention:quince | 0 | No concerns | Some concerns | No concerns | No concerns | No concerns | No concerns | Moderate | ["Reporting bias"] |
| no_intervention:sham_acupressure | 0 | No concerns | Some concerns | No concerns | Some concerns | No concerns | No concerns | Low | ["Reporting bias","Imprecision"] |
| placebo:quince | 0 | No concerns | Some concerns | No concerns | No concerns | No concerns | No concerns | Moderate | ["Reporting bias"] |
| placebo:sham_acupressure | 0 | No concerns | Some concerns | No concerns | No concerns | Some concerns | No concerns | Low | ["Reporting bias","Heterogeneity"] |
| placebo:sham_acupuncture | 0 | No concerns | Some concerns | No concerns | No concerns | No concerns | No concerns | Moderate | ["Reporting bias"] |
| quince:sham_acupressure | 0 | No concerns | Some concerns | No concerns | No concerns | No concerns | No concerns | Moderate | ["Reporting bias"] |
| quince:sham_acupuncture | 0 | No concerns | Some concerns | No concerns | No concerns | No concerns | No concerns | Moderate | ["Reporting bias"] |
| sham_acupressure:sham_acupuncture | 0 | No concerns | Some concerns | No concerns | Some concerns | No concerns | No concerns | Low | ["Reporting bias","Imprecision"] |

**
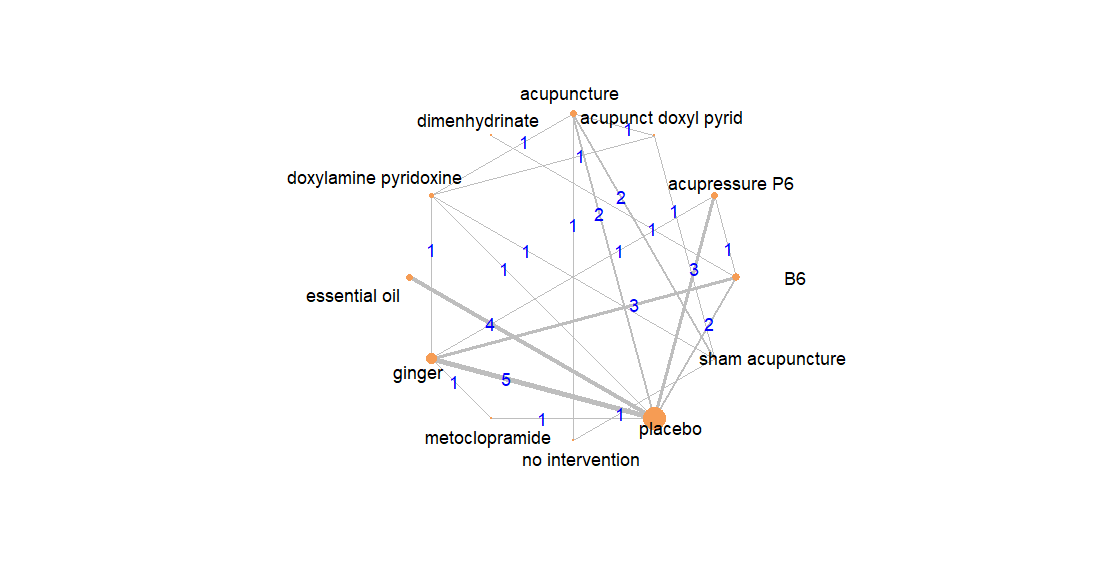
**

**Figure S4:** Network plot of efficacy analysis excluding single-study nodes and studies containing digitized outcomes

**
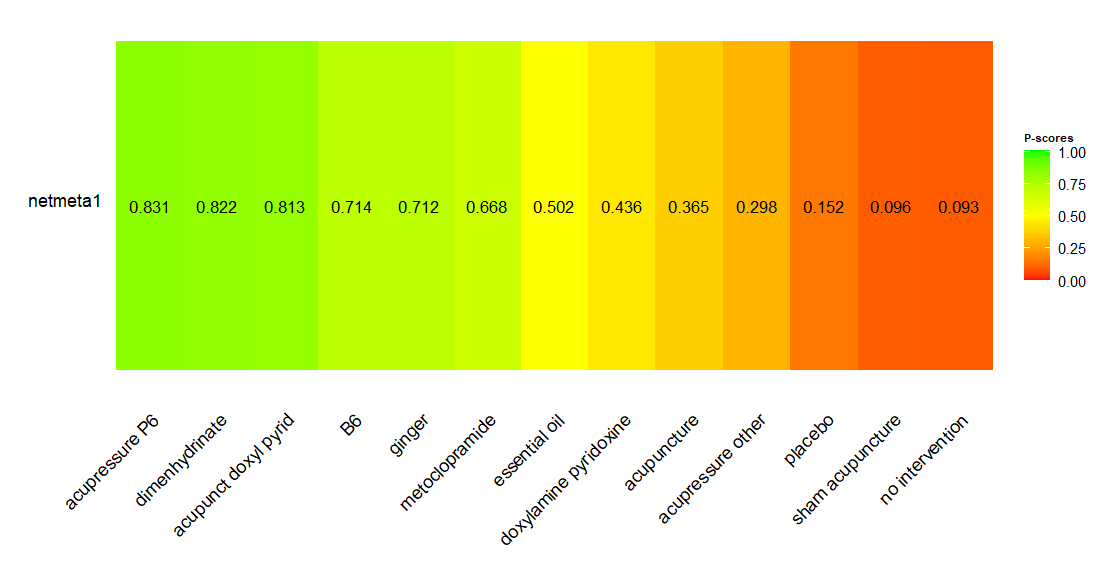
**

**Figure S5:** P-scores of interventions, efficacy analysis excluding single-study nodes and studies containing digitized outcomes

**
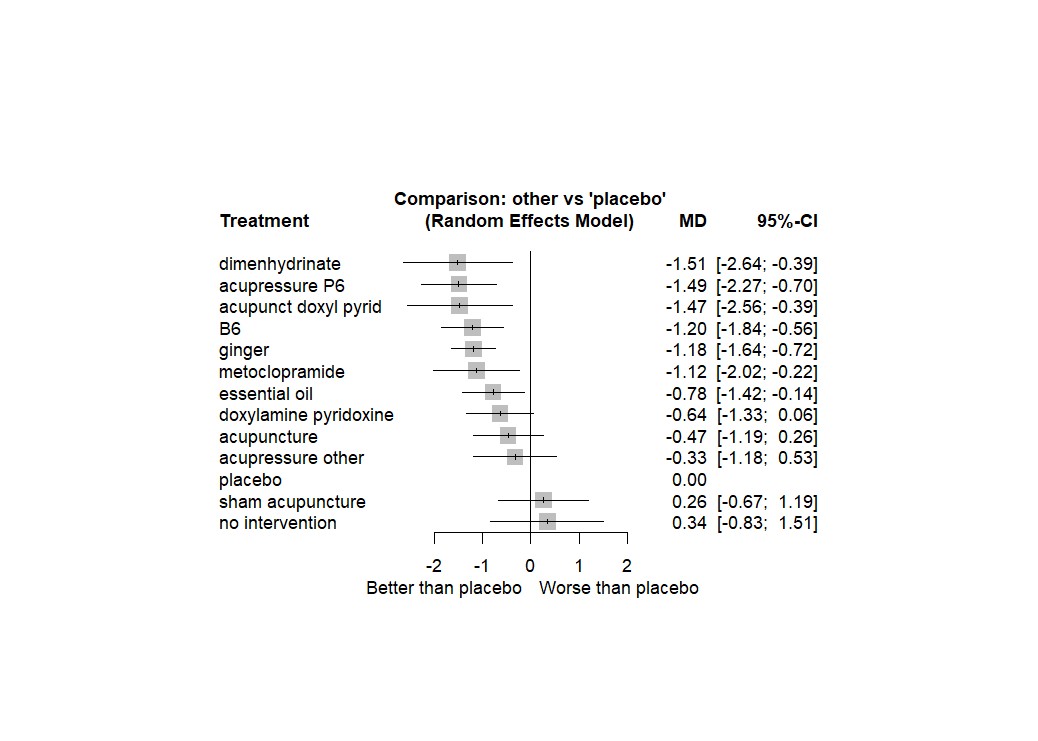
**

**Figure S6:** Forest plot with placebo reference, mean difference of NVP symptoms score reduction; efficacy analysis excluding single-study nodes and studies containing digitized outcomes

**
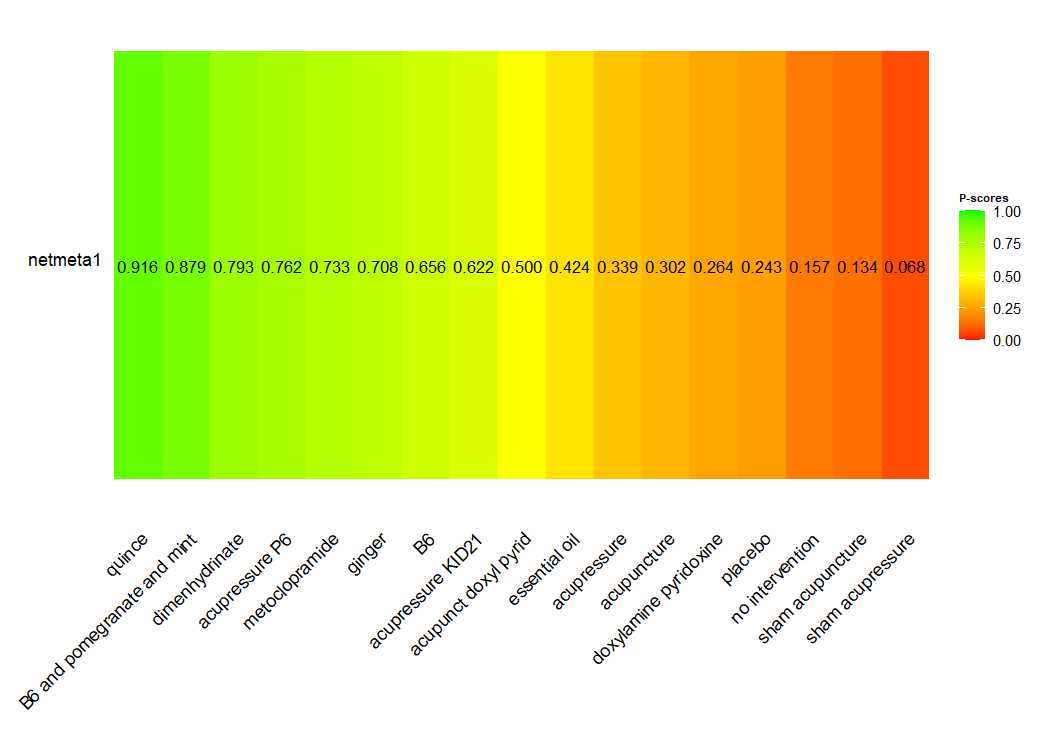
**

**Figure S7:** P-scores of interventions, SMD-based efficacy analysis

**
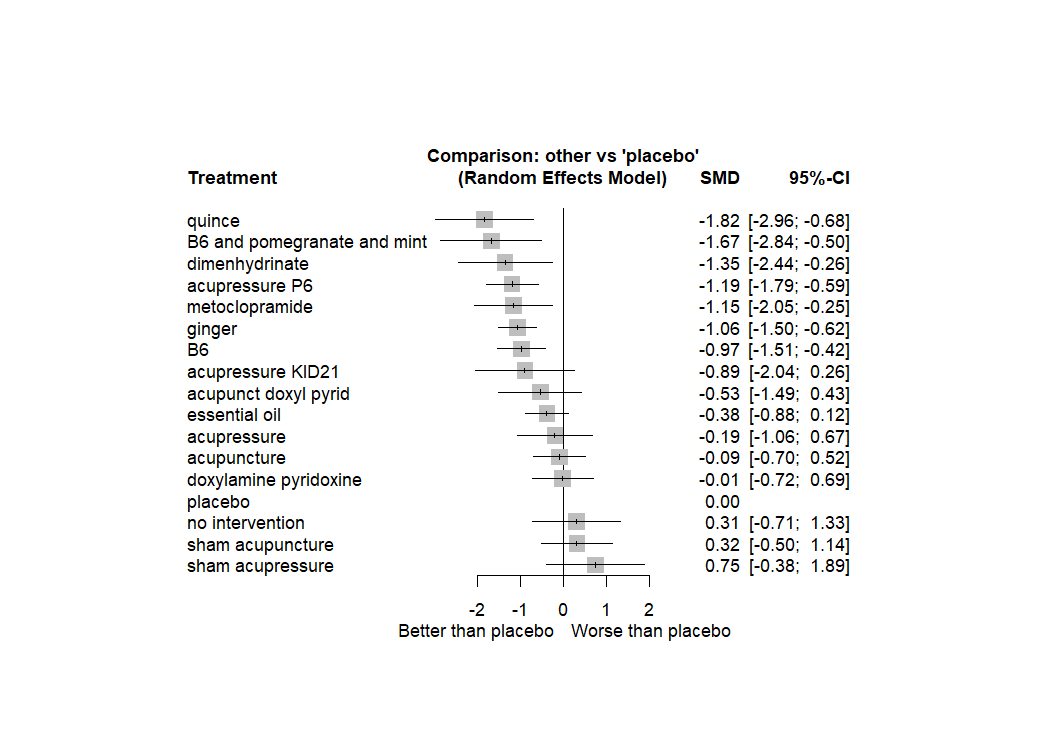
**

**Figure S8:** Forest plot with placebo reference, mean difference of NVP symptoms score reduction, SMD-based efficacy analysis

**REFERENCES**

1. Smith, C., C. Crowther, and J. Beilby, *Acupuncture to treat nausea and vomiting in early pregnancy: a randomized controlled trial.* Birth (Berkeley, Calif.), 2002. **29**(1): p. 1‐9.

2. Biswas, S.C., et al., *A single-masked, randomized, controlled trial of ginger extract in the treatment of nausea and vomiting of pregnancy.* Journal International Medical Sciences Academy, 2011. **24**(4): p. 167-169.

3. Galeshi, M., et al., *A comparison of the effect of pressure on the KID21 (Youmen) and P6 (Neiguan) points on the severity of nausea and vomiting of pregnancy.* Journal of complementary & integrative medicine, 2020. **17**(2).

4. Adlan, A.S., K.Y. Chooi, and N.A. Mat Adenan, *Acupressure as adjuvant treatment for the inpatient management of nausea and vomiting in early pregnancy: A double-blind randomized controlled trial.* J Obstet Gynaecol Res, 2017. **43**(4): p. 662-668.

5. Pasha, H., et al., *Study of the effect of mint oil on nausea and vomiting during pregnancy.* Iranian Red Crescent Medical Journal, 2012. **14**(11): p. 744-747.

6. Abdolhosseini, S., et al., *Effects of pomegranate and spearmint syrup on nausea and vomiting during pregnancy: A randomized controlled clinical trial.* Iranian Red Crescent Medical Journal, 2017. **19**(10).

7. Saberi, F., et al., *Acupressure and ginger to relieve nausea and vomiting in pregnancy: a randomized study.* Iran Red Crescent Med J, 2013. **15**(9): p. 854-61.

8. Yavari Kia, P., et al., *The effect of lemon inhalation aromatherapy on nausea and vomiting of pregnancy: a double-blinded, randomized, controlled clinical trial.* Iran Red Crescent Med J, 2014. **16**(3): p. e14360.

9. Firouzbakht, M., et al., *Comparison of ginger with vitamin B6 in relieving nausea and vomiting during pregnancy.* Ayu, 2014. **35**(3): p. 289-93.

10. Mohammadbeigi, R., et al., *Comparing the effects of ginger and metoclopramide on the treatment of pregnancy nausea.* Pakistan Journal of Biological Sciences, 2011. **14**(16): p. 817-820.

11. Fróes, N.B.M., et al., *Effects of auriculotherapy on nausea and vomiting in pregnant women: A randomized clinical trial.* Complement Ther Clin Pract, 2024. **55**: p. 101847.

12. Safajou, F., et al., *The Effect of Combined Inhalation Aromatherapy with Lemon and Peppermint on Nausea and Vomiting of Pregnancy: A Double-Blind, Randomized Clinical Trial.* Iran J Nurs Midwifery Res, 2020. **25**(5): p. 401-406.

13. Yilmaz, M.P., S. Yazici, and I. Yilmaz, *Effect of Acupressure at PC6 on Nausea and Vomiting During Pregnancy: a Randomized Controlled Trial.* Journal of acupuncture and meridian studies, 2023. **16**(3): p. 89‐94.

14. Chittumma, P., K. Kaewkiattikun, and B. Wiriyasiriwach, *Comparison of the effectiveness of ginger and vitamin B6 for treatment of nausea and vomiting in early pregnancy: a randomized double-blind controlled trial.* Chotmaihet thangphaet [Journal of the Medical Association of Thailand], 2007. **90**(1): p. 15‐20.

15. Solt Kirca, A. and D. Kanza Gul, *Effects of Acupressure Applied to P6 Point on Nausea Vomiting in Pregnancy: A Double-Blind Randomized Controlled.* Alternative therapies in health and medicine, 2020. **26**(6): p. 12-17.

16. Basirat, Z., et al., *The effect of ginger biscuit on nausea and vomiting in early pregnancy.* Acta Medica Iranica, 2009. **47**(1): p. 51-56.

17. Negar, et al., *Auriculotherapy as a means of managing nausea and vomiting in pregnancy: A double-blind randomized controlled clinical trial.* Complementary therapies in clinical practice, 2020. **40**: p. 101177.

18. Jamigorn, M. and V. Phupong, *Acupressure and vitamin B6 to relieve nausea and vomiting in pregnancy: a randomized study.* Arch Gynecol Obstet, 2007. **276**(3): p. 245-9.

19. Babaei, A.H. and M.H. Foghaha, *A randomized comparison of vitamin B6 and dimenhydrinate in the treatment of nausea and vomiting in early pregnancy.* Iranian journal of nursing and midwifery research, 2014. **19**(2): p. 199‐202.

20. Sharifzadeh, F., et al., *A comparison between the effects of ginger, pyridoxine (vitamin B6) and placebo for the treatment of the first trimester nausea and vomiting of pregnancy (NVP).* J Matern Fetal Neonatal Med, 2018. **31**(19): p. 2509-2514.

21. Joulaeerad, N., et al., *Effect of aromatherapy with peppermint oil on the severity of nausea and vomiting in pregnancy: A single-blind, randomized, placebo-controlled trial.* Journal of Reproduction and Infertility, 2018. **19**(1): p. 32-38.

22. Koren, G., et al., *Demonstration of early efficacy results of the delayed-release combination of doxylamine-pyridoxine for the treatment of nausea and vomiting of pregnancy.* BMC Pregnancy Childbirth, 2016. **16**(1): p. 371.

23. Wu, X.K., et al., *Acupuncture and Doxylamine-Pyridoxine for Nausea and Vomiting in Pregnancy : a Randomized, Controlled, 2 × 2 Factorial Trial.* Annals of internal medicine, 2023. **176**(7): p. 922‐933.

24. Jafari-Dehkordi, E., et al., *Comparison of quince with vitamin B6 for treatment of nausea and vomiting in pregnancy: A randomised clinical trial.* Journal of Obstetrics and Gynaecology, 2017. **37**(8): p. 1048-1052.
